# Cowpea (*Vigna unguiculata* L. Walp.) Metabolomics: Osmoprotection as a Physiological Strategy for Drought Stress Resistance and Improved Yield

**DOI:** 10.3389/fpls.2017.00586

**Published:** 2017-04-20

**Authors:** Piebiep Goufo, José M. Moutinho-Pereira, Tiago F. Jorge, Carlos M. Correia, Manuela R. Oliveira, Eduardo A. S. Rosa, Carla António, Henrique Trindade

**Affiliations:** ^1^Centre for the Research and Technology of Agro-Environment and Biological Sciences, Universidade de Trás-os-Montes e Alto DouroVila Real, Portugal; ^2^Plant Metabolomics Laboratory, Instituto de Tecnologia Química e Biológica António Xavier, Universidade Nova de LisboaOeiras, Portugal; ^3^Unidade de Biotecnologia e Recursos Genéticos, Instituto Nacional de Investigação Agrária e VeterináriaOeiras, Portugal

**Keywords:** drought, metabolome, cowpea, osmotic adjustment, gas exchange, chlorophyll fluorescence, metabolite profiling, adaptation

## Abstract

Plants usually tolerate drought by producing organic solutes, which can either act as compatible osmolytes for maintaining turgor, or radical scavengers for protecting cellular functions. However, these two properties of organic solutes are often indistinguishable during stress progression. This study looked at individualizing properties of osmotic adjustment vs. osmoprotection in plants, using cowpea as the model species. Two cultivars were grown in well-watered soil, drought conditions, or drought followed by rewatering through fruit formation. Osmoadaptation was investigated in leaves and roots using photosynthetic traits, water homoeostasis, inorganic ions, and primary and secondary metabolites. Multifactorial analyses indicated allocation of high quantities of amino acids, sugars, and proanthocyanidins into roots, presumably linked to their role in growth and initial stress perception. Physiological and metabolic changes developed in parallel and drought/recovery responses showed a progressive acclimation of the cowpea plant to stress. Of the 88 metabolites studied, proline, galactinol, and a quercetin derivative responded the most to drought as highlighted by multivariate analyses, and their correlations with yield indicated beneficial effects. These metabolites accumulated differently in roots, but similarly in leaves, suggesting a more conservative strategy to cope with drought in the aerial parts. Changes in these compounds roughly reflected energy investment in protective mechanisms, although the ability of plants to adjust osmotically through inorganic ions uptake could not be discounted.

## Introduction

Plants usually acclimate or adapt to marginal environments by engaging multiple protective mechanisms. Water is typically the most limiting resource to plant growth and productivity, making drought one of the most deleterious abiotic stresses with respect to fitness and survival (Pinheiro and Chaves, 2011; Simova-Stoilova et al., 2015; Gagné-Bourque et al., 2016). A range of adaptive features has been observed in plants exposed to drought stress, based on the concepts of escape, avoidance, and tolerance.

Plants “escape” drought by altering phenological development, often modifying the duration of a specific growth stage (Agbicodo et al., 2009). Avoidance mechanisms are primarily morphological and physiological adjustments to withstand water deficit while maintaining relatively high tissue moisture, and include increased root density or depth (Sicher et al., 2012), decreased stomatal and lenticular conductance, reduced leaf area, increased leaf waxiness and thickness (Singh and Raja Reddy, 2011), and leaf rolling or folding to minimize evapotranspiration (Fatokun et al., 2012; Hall, 2012). Tolerance traits maintain tissue hydrostatic pressure, primarily through osmotic adjustments, which results from compatible organic solute synthesis and accumulation in the cytoplasm and influx of mineral solutes into the vacuoles (Warren et al., 2011; Khan et al., 2015; Blum, 2017). Compatible solutes are typically hydrophilic and can replace water molecules on protein and membrane surfaces, eventually raising cellular osmotic pressure and increasing the water potential gradient between soil and roots, thereby allowing continued water influx via osmosis. Structural modifications of cell walls and membranes or stabilization of cellular structures also confer tolerance (Lugan et al., 2010; Jin et al., 2016). Stress resistance often depends on the ability of plants to use these mechanisms independently or jointly to minimize the negative consequences of water limitations.

Several drought adaptive mechanisms have been described in cowpea (*Vigna unguiculata* L. Walp.), which is reputed to be the most drought- and heat-resistant crop in semi-arid Africa. Cowpea supports millions of people in the tropics and subtropics, and is currently the focus of active breeding research for combating poverty in developing countries, in light of rising global temperatures and water scarcity (Hall, 2012). Cowpea drought resistance affects virtually all growth stages, and hydraulic responses have a genetic component (Muchero et al., 2008), which makes the species an interesting model for investigating the basis of drought adaptation.

Some cowpea varieties escape terminal drought by flowering 12 days earlier on average, whereas others can remain green for weeks without irrigation and only flower when favorable climatic conditions are re-established (Fatokun et al., 2012). Cowpea is fairly unusual among crops in that it exhibits very limited changes in leaf water content under extreme drought; this isohydric behavior has been associated with three avoidance mechanisms which are stomata closure, paraheliotropism, and high root hydraulic conductivity (Agbicodo et al., 2009).

The role of osmoadaptation in cowpea has been controversial, In some cultivars under water stress, fast and significant changes in proline favoring osmotic adjustment have been reported (Hamidou et al., 2007; Costa et al., 2011). In other cultivars, proline scarcely accumulated or appeared several days after irrigation ceased (Singh and Raja Reddy, 2011; Shui et al., 2013). Because of these variations, metabolic changes in cowpea under water deficit have mostly been linked to a stress-induced starvation injury, rather than a beneficial response. However, these late responses may be more specific and could be related to mechanisms induced by stomatal and non-stomatal photosynthesis limitations, and aimed at protecting the photosynthetic apparatus against reactive oxygen species. In general, atmospheric CO_2_ diffuses through stomata to the intercellular spaces, then across the mesophyll to the carboxylation sites. The limitations to CO_2_ assimilation imposed by stomatal closure (i.e., stomatal limitation of photosynthesis) in leaves during water stress may lead to an imbalance between electron generation at photosystem II (PSII) and electron requirements for photosynthesis. In turn, this could lead to overexcitation and subsequent photo-inhibitory damage of PSII reaction centers from mesophyll and biochemical limitations of photosynthesis. However, photo-inhibition may be prevented by processes such as non-photochemical quenching, thermal deactivation, and electron transport to O_2_, thus leading to photorespiration and/or Mehler peroxidise reactions (Pinheiro and Chaves, 2011; Sánchez-Martín et al., 2015). These protective mechanisms may disrupt cell homeostasis and may involve metabolic changes, such as the induction of compounds with antioxidant and chelating activities (Nakabayashi et al., 2014; Pan et al., 2016).

Moreover, studies of drought tolerance in cowpea have focused on proline and ignored possible changes in other compatible metabolites. However, there is increasing evidence that the osmolytes that play major roles in stress tolerance are specific to species and even varieties (Sanchez et al., 2012; Li et al., 2015; Obata et al., 2015). Effects of drought on a broader array of cowpea metabolites have not been examined, so it is not known which compounds contribute the most to osmoadaptation. A metabolite profiling approach with the appropriate design (Lisec et al., 2006; Jorge et al., 2015; Chmielewska et al., 2016) and within the right discovery context could well-facilitate the identification of metabolic traits of drought resistance in cowpea.

Accordingly, the present study investigated the metabolic attributes of osmoregulation (osmotic adjustment vs. osmoprotection) in cowpeas. The study provides a detailed intertissue (leaves and roots) metabolic profiling analysis of a broad range of primary and secondary metabolites in cowpea, including elemental solutes. By comparing drought and recovery responses at different developmental stages, the study also sheds light on biochemical acclimation. Finally, by correlating grain yield with the identified metabolic biomarkers, the study provides an important empirical basis for improving drought resistance in marker-assisted breeding via altered metabolism. Despite the complexity of the drought response in cowpea, it was hypothesized that this approach, based on water restriction and rewatering, could better help distinguish the metabolic pathways active in stress perception and facilitate the selection of critical metabolites for drought tolerance in functional terms.

## Materials and methods

### Plant material and growth conditions

Two cowpea cultivars with contrasting responses to drought but with similar flowering times and grain yield reductions were used. Fradel seeds (accession Cp5051) were obtained from INIAV (Instituto Nacional de Investigação Agrária e Veterinária); and Pinhel seeds (accession Vg50) were kindly supplied by the Departamento de Genética e Biotecnologia of UTAD (Universidade de Trás-os-Montes e Alto Douro). The drought stress response in the cultivars is manifest in two discrete phenotypes, namely increased root length in Pinhel after exposure to stress, and increased stem diameter in Fradel upon rewatering (**Figure 12A**).

The trials were conducted using 11-L plastic pots (25.5 cm in height) with drainage holes. Each pot was filled with 12 Kg of an 8:3 (w:w) mixture of soil and sand (Supplementary Table 1). Prior to sowing, the pots were thoroughly soaked with water to ensure 100% field capacity, which was the weight of the pots with saturated soil. To determine 0% field capacity, saturated soil was oven-dried to a constant weight. Plants were grown in a greenhouse at UTAD (N 41°17′7.28″, W 7°44′36.83″) with mean 43.55 ± 5.47/19.07 ± 2.88 °C day/night air temperatures, and a 16-h photoperiod, with photosynthetic photon flux density (PPFD) of up to 1701.42 ± 50.14 μmol m^−2^ s^−1^ (Supplementary Figure 1).

### Sowing and experimental design

The layout of the experiment was a factorial design consisted of three adjacent blocks, one for each sampling event. Within each block, the pots were arranged in a randomized complete block design with four or six replicate pots per cultivar and treatment. The pots were sown on 18 May with four seeds at 80% field capacity, and thinned to two plants 15 days later.

**Figure 1:**
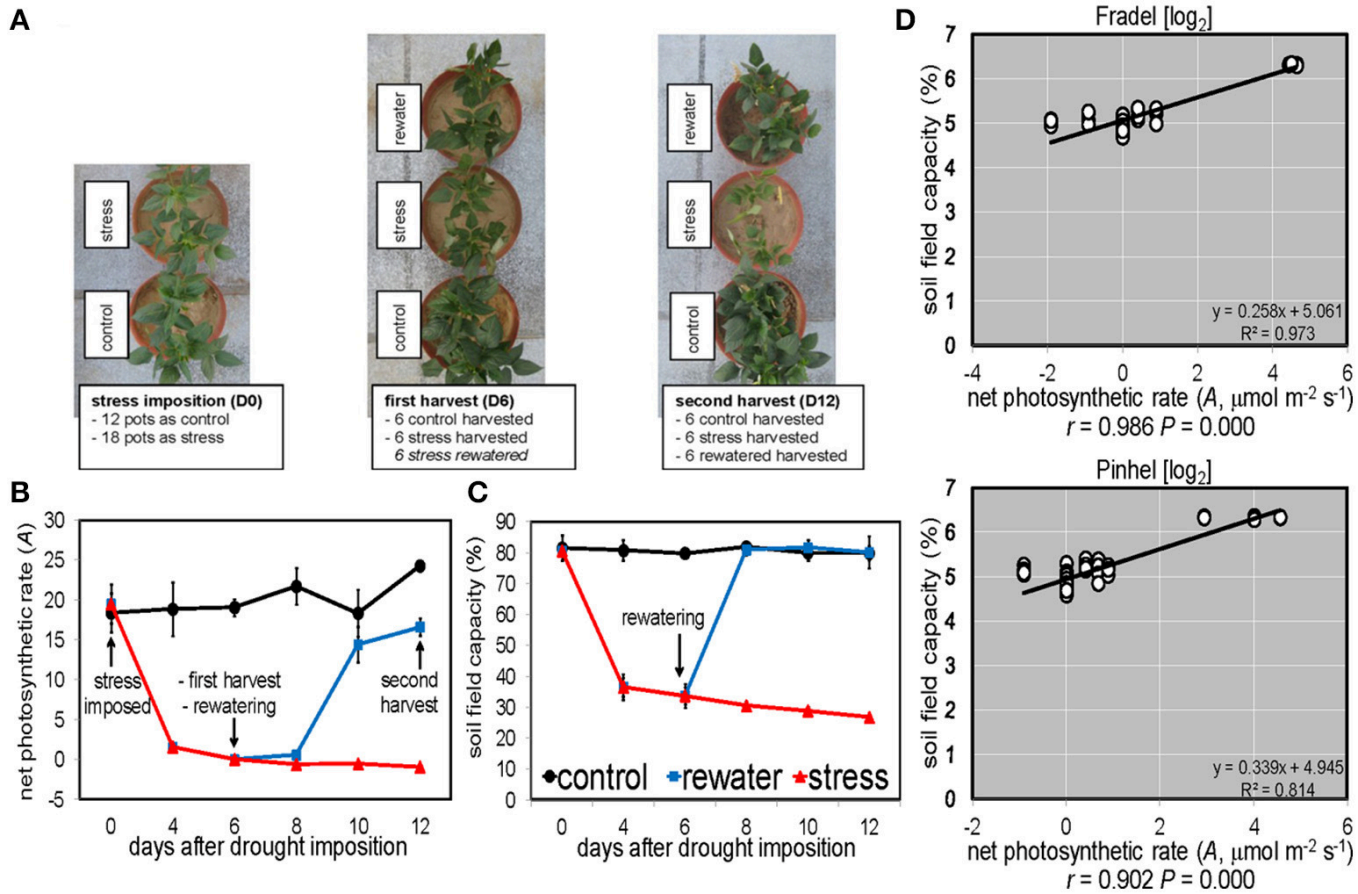
**Experimental layout and sampling scheme. (A)** Top view of representative Pinhel plants at key points during the experiment. **(B)** Vegetative plants were harvested on day 6 (net photosynthetic rate *A* = 0 in stressed plants) and reproductive plants on day 12 (*A* = control). **(C)** Forty-day-old control plants were maintained at 80% field capacity. Stressed plants were not watered for 6 days, when a cohort was rewatered (*n* = 6 with two plants per pot; error bars = SD). **(D)** Logarithmic scaling of the relationship between field capacity and *A* of plants exposed to water deficit from day 0 until 12. *R*^2^, coefficient of determination; *r*, Pearson correlation coefficient; *P*, probability. Another set of plants (*n* = 4) was grown till grain maturity (day 40).

Based on results from a pilot study to establish drought regimes, the drought experiment started 40 days after sowing, when plants began branching (Figure 1). The experiment focused on the progressive development of stress, as well as on two steady-state situations (e.g., Sicher et al., 2012), the first in which the plants were significantly stressed during the vegetative stage, but not lethally damaged (first block—first harvest), and the second in which stressed plants recovered during the reproductive stage upon rewatering (second and third blocks—second and third harvests). The first block included well-watered and drought-stressed treatments with six replicate pots. The well-watered control was maintained at 80% field capacity (by weighing the pots two times per day and adding the amount of water equal to the loss in weight), while in the other treatment plants were progressively stressed by stopping watering until the net photosynthetic rate (*A*) reached 0 on day 6 (D6), when plants were harvested (vegetative stage). The second block included well-watered, drought-stressed, and rewatered treatments, also with six replicate pots. When *A* reached 0 on D6, half of the plants were rewatered to return their soil to 80% field capacity and allowed to grow until *A* matched the control on day 12 (D12), when plants were harvested (reproductive stage). The third block mirrored the second one, but with four replicates and pots weighed every 2 days after day 12; plants were grown until grain maturity (D40), which provided a more detailed assessment of the treatment effects on yield and yield-related parameters.

At harvest, plants were uprooted before midday, washed gently with water to remove soil from roots, which were rinsed three times with 10 mM MES + KOH buffer (pH 6.5) to remove biological contaminants, and then blotted dry. Homogeneous healthy leaves of the same sizes and ages (three to five youngest fully expanded leaves) were removed from stems, and roots were cut off. Samples from both plants in each pot were pooled, flash frozen in liquid nitrogen, and stored at −80°C until analysis.

### Gas exchange and chlorophyll fluorescence measurements

Leaf gas exchange measurements were performed on the uppermost fully expanded leaf of each plant in the second block every other day, using a portable infrared gas analyser (LCpro+, ADC, Hoddesdon, England) under the same conditions of growth. Net photosynthetic (CO_2_ assimilation) rate (*A*), stomatal conductance (g_s_), ratio between intercellular and atmospheric CO_2_ concentration (Ci/Ca), and transpiration rate (E) were determined. Concurrently, chlorophyll *a* fluorescence was measured *in situ* using a pulse amplitude modulation system (FMS 2, Hansatech Instruments, Norfolk, England). Minimum fluorescence (F_0_) was measured in 30 min dark-adapted leaves by applying a weak modulated light, and maximum fluorescence (F_m_) was measured after applying a 0.7 s saturating pulse of white light (>1,500 μmol m^−2^ s^−1^). The maximum quantum yield of photosystem II was calculated as F_v_/F_m_ = (F_m_− F_0_)/ F_m_ (Roháček, 2002). Following F_v_/F_m_ estimation, after a 20-s exposure to actinic light (1,500 μmol m^−2^ s^−1^), light-adapted steady-state fluorescence yield (F_s_) was averaged over 2.5 s, followed by exposure to saturating light (15,000 μmol m^−2^ s^−1^) for 0.7 s to establish F'_m_. The sample was then shaded for 5 s with a far-red light source to determine F'_0_. From these measurements the electron transport rate (ETR), photochemical quenching (qP), non-photochemical quenching (NPQ), effective quantum yield of PSII (Fv′/Fm′), quantum efficiency of PSII (Φ_PSII_ = ΔF/F'_m_ = (F'_m_−F_s_)/F'_m_), and the fraction of PPFD neither utilized in photochemistry nor dissipated thermally (P_E_) were calculated as described by Maxwell and Johnson (2000).

### GC-TOF-MS profiling of primary metabolites

Primary metabolites were extracted and derivatized following the well-established method of Lisec et al. (2006). Frozen tissue was ground in liquid nitrogen to a fine powder with a mortar and pestle, transferred to a tube (100 mg) with 1.4 mL methanol, vortexed for 2 s, and 60 mL ribitol (0.2 mg/mL) added as internal standard; the samples and consumables were kept on ice to prevent thawing. The mixtures were shaken in a ThermoMixer at 950 rpm for 10 min at 70°C; tubes were opened occasionally to release pressure, and then centrifuged for 10 min at 11,000 × g at 4°C. The supernatant was collected, and the polar fraction was partitioned in 0.75 mL chloroform with 1,400 mL water. After centrifugation at 2,200 × g for 15 min, 150 mL of the upper layer was dried in a centrifugal concentrator overnight. In a derivatization reaction, the dry residue was dissolved in 40 μL of 20 mg/mL methoxyamine hydrochloride in pyridine at 37°C for 2 h and then ultrasonicated at 37°C for 30 min in 70 μL N-methyl-N-(trimethylsilyl)trifluoroacetamide that contained 20 μL/mL of a retention time index standard mixture composed of fatty acid methylesters in chloroform (0.4 mL/mL).

An aliquot (1 μL) of derivatized sample was injected into an Agilent 7890A gas chromatography (GC) (San Joseph, MI) in both splitless and split (1:30) modes at 230°C. Helium was used as the carrier gas with a flow rate of 2 mL/min, and separation was accomplished using a DB-35MS column (30 m × 0.32 mm, 0.25 μm; Agilent Technologies). The gradient program was 2 min at 85°C, and then 15°C/min to 360°C. Column eluates were introduced into a Pegasus HT time-of-flight mass spectrometer (TOF-MS, LECO, San Joseph, MI) through an electron ionization source operating in negative ion mode and ionized using a filament bias current of 70 eV. The transfer line and ion source were all held at 250°C. Mass spectra were collected from m/z 50 to 500.

Acquired chromatograms and mass spectra were evaluated using the built-in deconvolution algorithm of ChromaTOF®, and TagFinder (Luedemann et al., 2008). Compounds were identified by comparing mass spectral and retention time indices to those in the Golm metabolome database (www.mpimp-Golm.mpg.de/csbdb/gmd/gmd.html). Metabolite levels were determined as relative abundances normalized to the signal intensity of the internal standard ribitol and sample fresh weight (Supplementary Table 2).

### LC-DAD profiling of secondary metabolites

Secondary metabolites, primarily phenolics, were extracted and characterized using liquid chromatography/photodiode array detector (LC-DAD), based on the method of Hachibamba et al. (2013). Naringin (20 μL) was added to 40 mg of sample as a peak reference, and phenolics were extracted with 1 mL of 70% methanol and continuously shaken for 30 min at ~25°C. Following centrifugation at 10,000 × g for 15 min, the resulting supernatants were passed through a 0.2 μm Spartan 13/0.2 RC Whatman filter.

Subsequently, 10 μL of solution was analyzed and quantified using a Gilson LC (Villers-le-bel, France) equipped with a Finnigan/Surveyor 81401 DAD (Thermo Electron, San Jose, CA). Samples were separated using a C18 column (150 × 4.6 mm, 2.6 μm; Phenomenex, Torrance, CA) at 25°C. The mobile phase consisted of 0.1% aqueous trifluoroacetic acid as solvent A and 0.1% trifluoroacetic acid in acetonitrile as solvent B, with a linear gradient elution at a flow rate of 1 mL/min as follows: 8–10% B (0–10 min), 10–20% B (10–35 min), 20–20% B (35–50 min), 20–45% B (50–60 min), and 45–8% B (60–80 min). Proanthocyanidins, phenolic acids, flavonoids, and anthocyanins were monitored at 280, 320, 360, and 520 nm, respectively.

Data acquisition and processing were performed with Xcalibur 2.0 and compounds were identified by comparing their retention times and spectroscopic data with those of authentic standards. In the absence of a standard, identification was made by matching DAD signals and comparing elution profiles to those reported in the literature. Quantification using peak areas was based on response factors (Supplementary Table 3), after normalization with the internal standard naringin and sample fresh weight.

### Estimation of water relations

The water content of shoots, leaves, and roots was estimated after oven drying. Immediately after completing the gas exchange analyses, the measured leaves were removed for leaf water potential (ψ_w_) determination. A different set of leaves was used for the relative water content (RWC) and leaf solute (osmotic) potential (ψ_s_), since the ψ_w_ measurements were followed by leaf destruction. RWC was calculated as the weight ratio between the leaf water content *in situ* and the completely turgid water content after rehydration (Kwasniewski et al., 2016). ψ_w_ was determined using a pressure bomb Model 1000 (PMS Instruments Co., Albany, OR) (Mendes et al., 2001; Warren et al., 2011). Immediately after the leaves were excised from the plant by using an oblique razor from 2 cm from the petiole, they were enveloped in the instrument chamber. The rate of increase of the inlet of gas into the cylinder was maintained constant at 0.02 MPa s^−1^, until the xylem sap appeared at cut surface of the petiole; the readings were recorded in Bars and expressed in MPa. ψ_s_ was determined with a thermocouple psychrometer (SC-10, Decagon Devices Inc., Pullman, WA) in punched leaf discs (ca. 0.70 cm^2^ each) frozen in liquid nitrogen, and kept at −80°C (Mendes et al., 2001). Frozen leaf discs were placed in psychrometer steel cups and left to equilibrate for 2 h before measurements and readings taken after 2 min. To convert the μV output to corresponding pressure values (MPa), a standard regression curve was obtained with eight known KCl concentrations in the expected ψ_s_ range. The pressure potential (ψ_p_) was simply calculated by the equation: water potential—solute potential. In order to estimate osmotic adjustment, the obtained ψ_s_ values were corrected to full turgor with the RWC value of the corresponding sample ($\psi_{\pi }^{100}$ = ψ_s_ × RWC/100). The degree of adjustment was calculated as the absolute difference between $\psi_{\pi }^{100}$ values of the different treatments.

### Determination of elemental solutes

C and N were determined from 0.2 g of freeze-dried sample using the Dumas' combustion principle with Primacs SNC-100 (Skalar Analytical B.V, Breda, Netherlands). P and K were extracted from 0.3 g sample by digestion with H_2_SO_4_, followed by Skalar San^++^ segmented flow analysis and Jenway PFP7 flame photometry (Essex, UK), respectively.

### Statistical analyses and data presentation

Shapiro-Wilk's and Levene's tests conducted using SPSS 16.0 (SPSS Inc., Chicago, IL) indicated that the assumption of normal distribution and variance homogeneity was violated in most cases. Therefore, data were arcsine or log transformed before analyses. Data are mainly presented as means ± standard deviation (*SD, n* = 4–6) or as response ratios calculated as log fold-changes in contents between drought-stressed and well-watered, rewatered and drought-stressed, or rewatered and well-watered plants. ANOVA, MANOVA, and MANCOVA were performed using SPSS, and differences between means determined by Tukey's and Bonferroni's tests. Probabilistic principal component analyses (PCA) and Student's *t*-tests were performed using the statistical tool of MS/data-independent-acquisition (Tsugawa et al., 2015). Correlations were established by calculating Pearson coefficients, using the cor.prob function of R (www.R-project.org/). Parameters for which comparisons met a threshold of significance were chosen for further analysis using general linear regression modeling in EXCEL. Bonferroni correction was applied for multiple *t*-tests or Pearson's correlation. Differences between treatments were highlighted in heatmaps with hierarchical clustering (HCA, R package “pheatmap”) and in pathway maps according to the KEGG database (www.genome.jp/kegg/pathway.html).

## Results

### Effect of drought and rewatering on gas exchange and chlorophyll fluorescence

**Figure 2:**
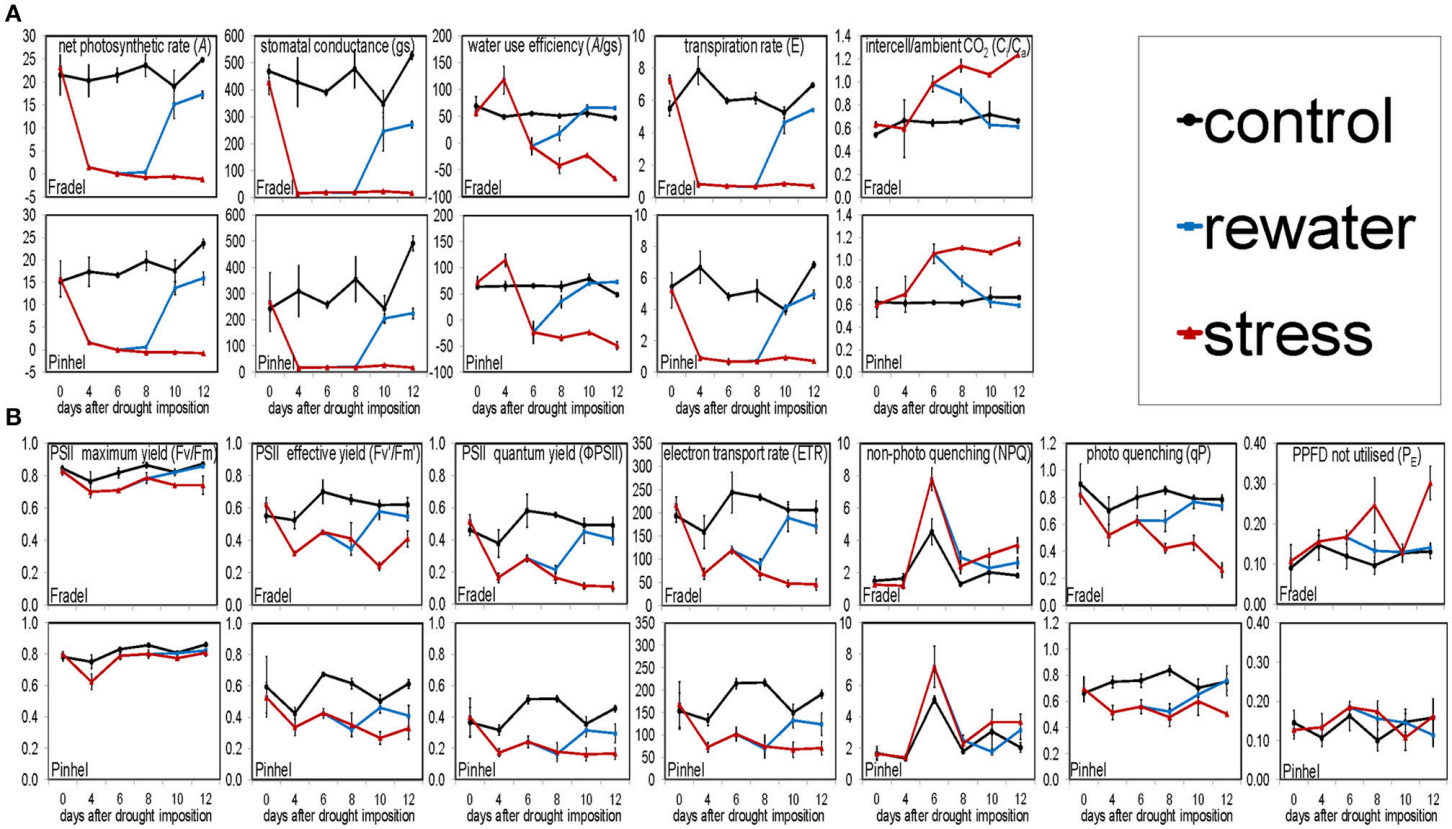
**Effects of drought stress and rewatering on gas exchange (A)** and chlorophyll fluorescence **(B)** parameters in cowpea. Lines indicate the well-watered control (black), drought-stressed (red), and rewatered plants (blue; *n* = 6; error bars = *SD* divided by 2 for better visibility).

Under drought (Figure 1A), *A* decreased with stress duration and reached 0 at D6 (Figure 1B), at a 34% field capacity (Figure 1C). Photosynthesis clearly depended on soil water status, with a linear relationship between *A* and field capacity (Figure 1D). Similar relationships were found for transpiration rate (E) and stomatal conductance (g_*s*_), but not for the other gas exchange and chlorophyll fluorescence parameters (not shown). *A* recovered to control levels (*P* < 0.05) after 4 days of rewatering (D10); however, plants were grown for another 2 days (D12) for equilibration. Pinhel had constitutively lower (*P* < 0.05) average g_*s*_ values than Fradel, 242.53 and 468.63 mmol m^−2^·s^−1^, respectively (Figure 2A). Under water stress, Pinhel exhibited a slower rate and lower reduction (*P* < 0.05) of Fv′/Fm′ (effective quantum yield of PSII) and qP (photochemical quenching coefficient) (Figure 2B), which suggested that Pinhel maintained more efficient photochemical reactions. To further test that inference, hierarchical clustering was performed, and demonstrated that the primary differences in responses to stress and recovery were in Fv/Fm (maximum quantum yield of PSII) and P_E_ (fraction of PPFD neither utilized in photochemistry nor dissipated thermally), which were correlated with gas exchange parameters in Fradel but not in Pinhel (Supplementary Figure 2; Supplementary Table 4). More specifically, Fv/Fm decreased in Fradel from D8 to D12 but not in Pinhel; and P_E_ increased in Fradel but not in Pinhel by the end of the experiment (*P* < 0.05; Figure 2B), suggesting that under more severe stress conditions, the capacity of protective mechanisms in Fradel could be exceeded.

### Effect of drought and rewatering on cowpea metabolome

GC-MS-based metabolite profiling identified 41 primary metabolites, including sugars (5), polyols (4), amino acids (18), amino acid derivatives (6), and organic acids (8) (Supplementary Table 2). Within the LC-DAD profiling dataset, 35 peaks with spectral characteristic of phenolic acids (15), flavonoids (17), and proanthocyanidins (3) were annotated (Supplementary Table 3; Supplementary Figure 3). Peaks matching no known structure (12) were labeled “unidentified” or “unknown,” for those absorbing at 290 and 435 nm, respectively.

MANOVA data (not shown) indicated that cultivar had no significant effect on metabolite content (*P* = 0.367). Instead, most differences could be attributed to spatial distribution, with sugars (0.39 vs. 0.28 on average), amino acids (0.58 vs. 0.36), and proanthocyanidins (31.96 vs. 21.15) predominantly observed in roots vs. leaves (*P* < 0.05), most likely owing to their higher energy demand for the assimilation of soil resources. Changes induced by the treatments were also larger than differences between cultivars, indicating little metabolic divergence between Fradel and Pinhel.

**Figure 3:**
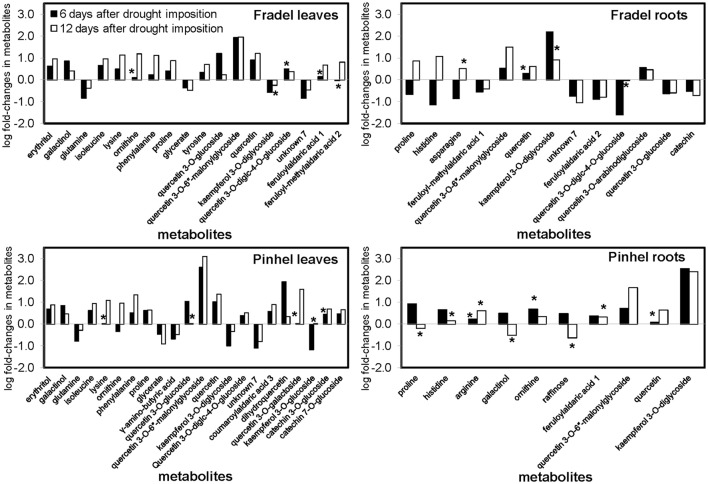
**Comparison of metabolite changes in cowpea in response to 6 and 12 days of drought stress**. Log fold-changes were determined relative to the control. Only metabolites that showed at least a 0.5-fold change at day 6 or day 12 are displayed. Metabolites marked with ^*^ were not statistically different between well-watered and drought-stressed conditions (*t*-test, *P* < 0.05).

**Figure 4:**
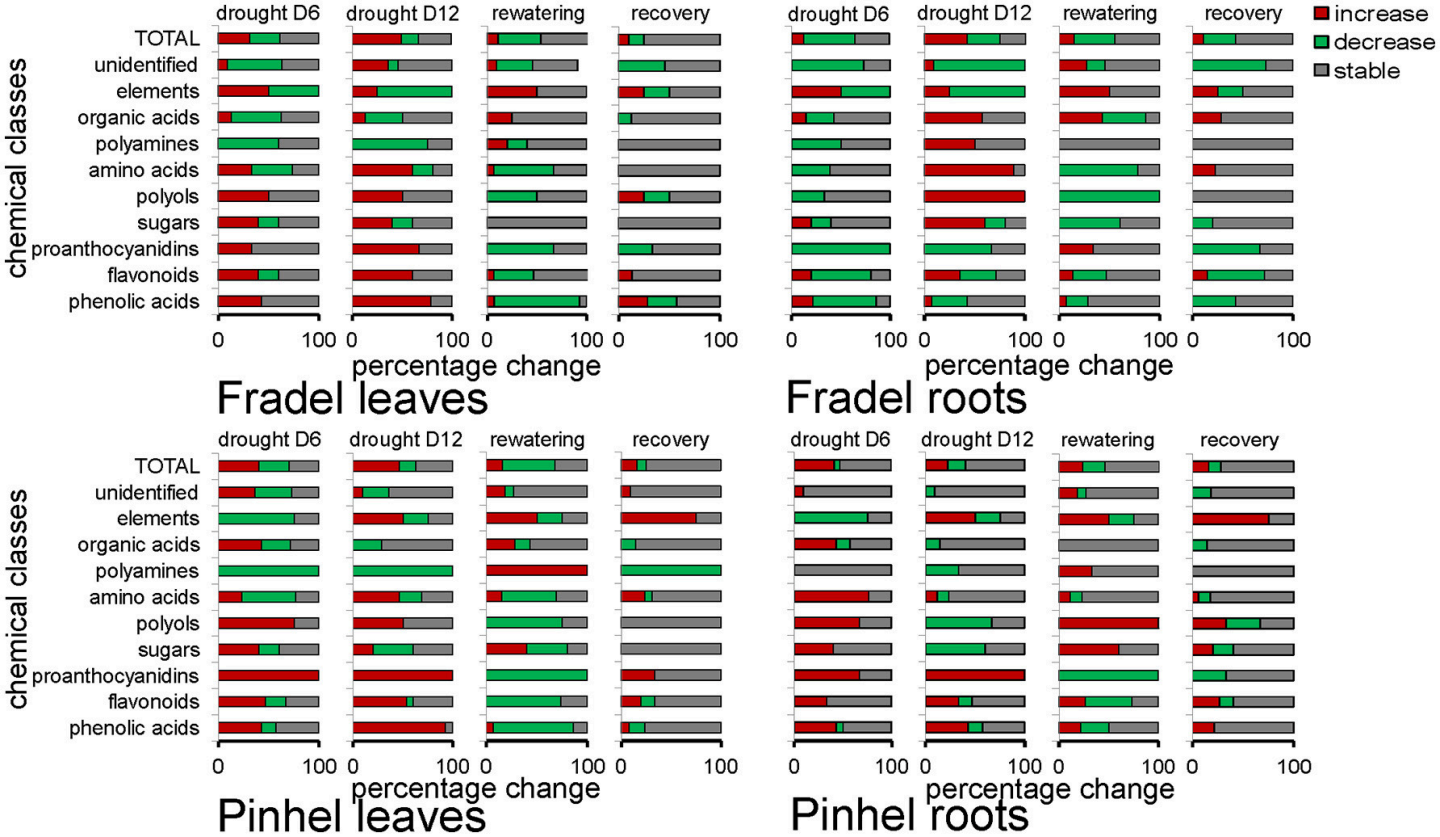
**Percentage (0–100% in the x-axis) of metabolites whose levels (***n*** = 4 to 6) were altered or remained unchanged (***P*** < 0.05; ***t***-test) after 6 days (drought D6 = stress relative to control) or 12 days (drought D12 = stress relative to control) of drought stress, and following 6 days of rewatering for plants stressed for 6 days (rewatering = rewatered relative to stress; recovery = rewatered relative to control)**. Metabolites were grouped by biochemical families, with 15 phenolic acids, 17 flavonoids, 3 proanthocyanidins, 5 sugars, 4 polyols, 18 amino acids, 6 polyamines and amino acid derivatives, 8 organic acids, 12 unidentified metabolites, and 4 elemental solutes. Within each bin, the proportions of compounds that increased, decreased, or held steady are coded red, green, and gray, respectively.

**Figure 5:**
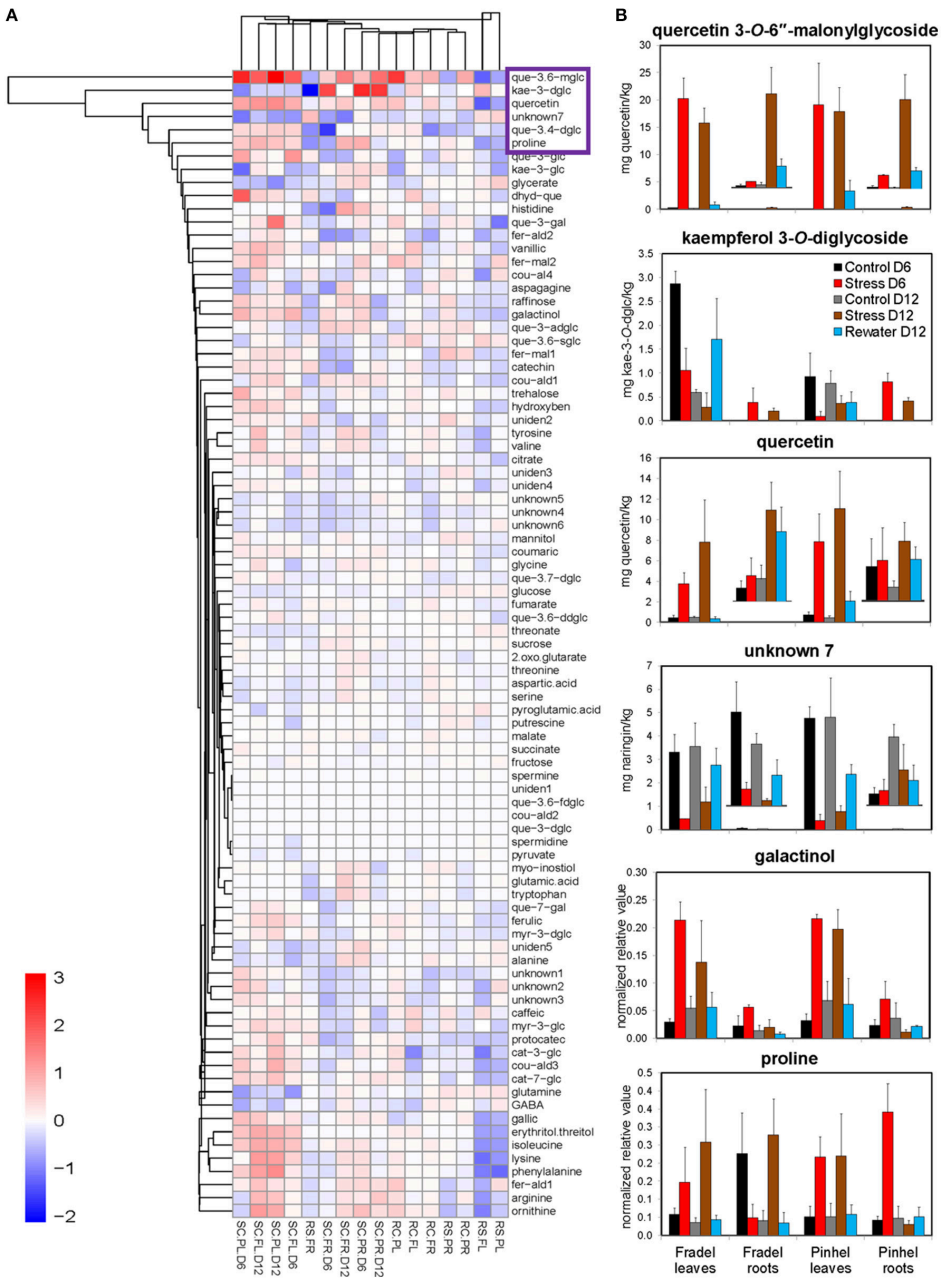
**Key metabolites involved in osmoadaptation in cowpea during a drought-rewatering course. (A)** Clustering used complete agglomeration and Manhattan distance. Each comparison is abbreviated and shown as a single column. The first two letters in the abbreviation indicate the treatments: SC = log(stress/control), RS = log(rewater/stress), and RC = log(rewater/control). The middle two letters indicate cultivars: PL for Pinhel leaves, PR for Pinhel roots, FL for Fradel leaves, and FR for Fradel roots. D indicates the duration of drought stress without rewatering: D6 and D12 for 6 and 12 days, respectively. The complete names for secondary metabolites can be retrieved from Supplementary Table 3. **(B)** Plots of the six drought-responsive compounds highlighted by all statistical tests, with five clustered in the purple box in the heatmap (*n* = 4 or 6; error bars = SD).

Drought responses during the vegetative (first harvest at D6) and reproductive (second harvest at D12) stages were compared using MANCOVA, keeping sampling time as a within-subject factor. Time and all it interacting factors were significant predictors of metabolite changes, *F*_(1, 2776)_ = 21.723, *P* = 0.000. The time × treatment interaction indicated that the influence of stress on metabolites was more prolonged over time, with overall increases and decreases in the responses of 42.11 and 19.74% of metabolites, respectively, between D6 and D12 (data not shown). The MANCOVA model used here in no way assumed that metabolites had identical response directions, but simply indicated with good confidence a cumulative effect of stress on the metabolome. In Fradel leaves, for example, levels of phenylalanine increased 0.22-fold at D6 but 1.12-fold at D12 (Figure 3). Figure 4 showed that not only qualitative, but also quantitative changes occurred in the metabolome over time. After 6 days of stress, 26, 31, and 10 metabolites increased (*P* < 0.05) in Fradel leaves, Pinhel leaves, and Fradel roots, respectively; after 12 days of stress, 41, 35, and 35 metabolites were increased. Conversely, 25, 23, and 43 metabolites decreased (*P* < 0.05) in the same organs at first harvest and were reduced to 14, 13, and 26 at second harvest. Pinhel roots were the exception in that fewer metabolites increased with stress progression (34–18), whereas more metabolites decreased (5–15). Interestingly, in most cases, the rate at which a metabolite changed in response to stress closely matched the rate at which it reverted upon rewatering. For example, after 12 days, proline levels in Pinhel stressed roots decreased 0.20-fold, whereas those in rewatered roots increased 0.23-fold (Figure 5B).

### Identification of drought-responsive metabolites

First, *t*-tests were used to identify metabolites with the greatest responses to drought stress. Only metabolites with a log fold-change of at least 0.5 relative to the control at D6 or D12 were plotted: 18 for Fradel leaves, 22 for Pinhel leaves, 13 for Fradel roots, and 10 for Pinhel roots (Figure 3). In the leaves, the largest changes were in quercetin 3-*O*-6″-malonylglycoside (tentative identification; 3.09-fold at D12 in Pinhel), and quercetin (1.37-fold at D12 in Pinhel) for secondary metabolites, and phenylalanine (1.33-fold at D12 in Pinhel), and ornithine (1.18-fold at D12 in Fradel) for primary metabolites. The levels of these compounds generally increased, with the exception of glutamine, glycerate, γ-aminobutyrate, kaempferol derivatives, and unknown7, which decreased at both harvests. Pronounced increases in roots included kaempferol 3-*O*-diglycoside (2.55-fold at D6 in Pinhel) and quercetin 3-*O*-6″-malonylglycoside (1.67-fold at D12 in Pinhel).

Second, a snapshot of drought-induced changes in the metabolome across harvesting times, cultivars, and organs was obtained using MANOVA. In stressed plants, levels of 63.15% of the 88 assessed compounds were different (*P* < 0.05) from those in control plants (Supplementary Table 5). Metabolites whose fold-change was greater than 0.5 were listed as discriminatory in decreasing order by absolute value: quercetin 3-*O*-6″-malonylglycoside (2.14-fold), quercetin (1.12-fold), erythritol (0.80-fold), unknown7 (−0.75-fold), galactinol (0.65-fold), proline (0.63-fold), phenylalanine (0.62-fold), and glycerate (−0.53-fold). A 0.57-fold-change was observed in isoleucine, but with *P* = 0.082.

**Figure 6:**
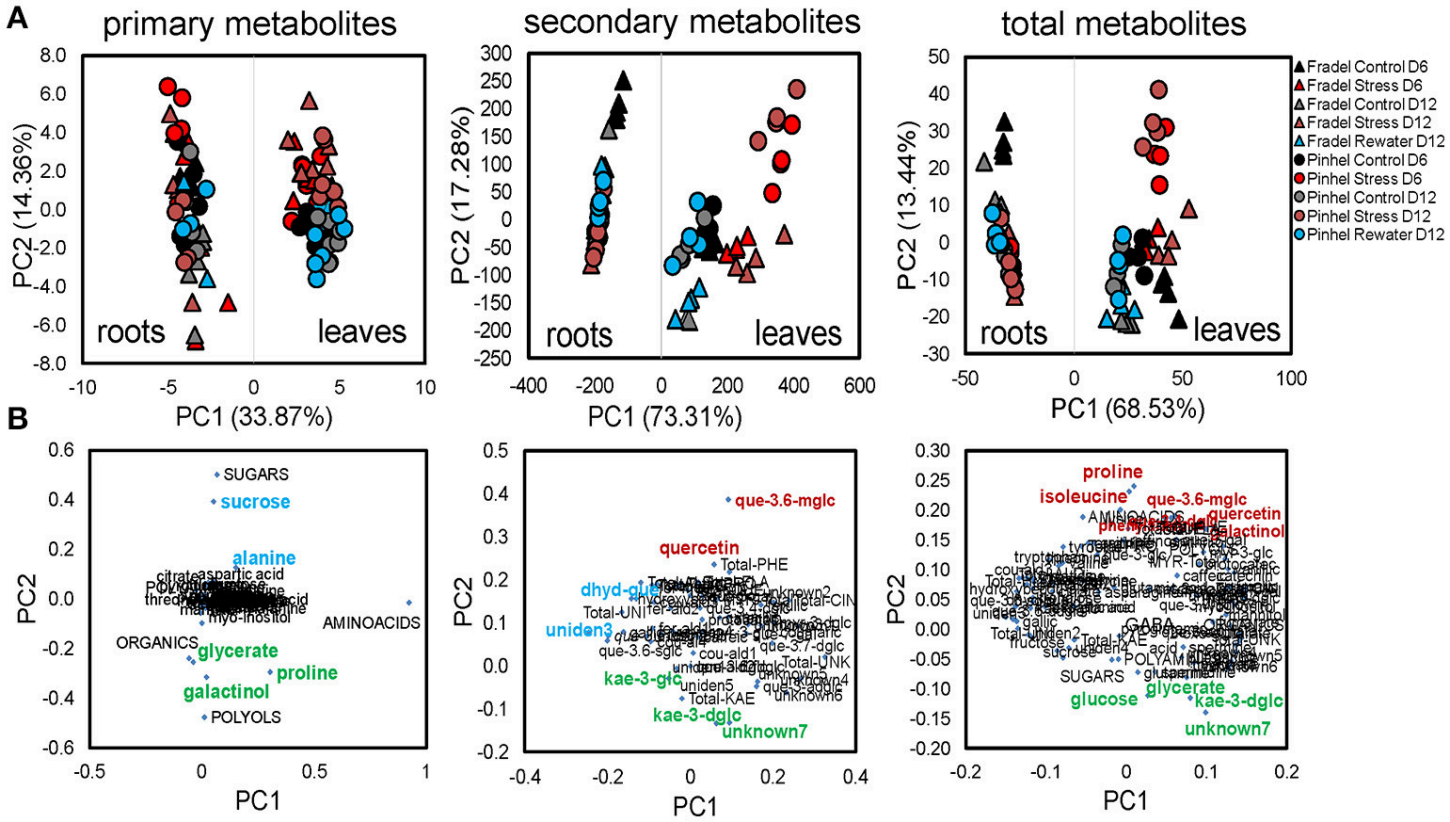
**PCA of metabolic differences among well-watered control, drought-stressed, and rewatered cowpea plants**. Shown are 104 variables: 41 GC-MS primary metabolites + 5 categories, and 35 LC-DAD secondary and 12 unidentified metabolites + 11 categories (*n* = 4–6). Data were square root-transformed with pareto scaling. **(A)** PCA scores with treatments as indicated in the legend. **(B)** Highest positive and negative loadings (Supplementary Table 6) associated with metabolic responses to stress/rewatering are tagged in red and green, respectively. Loadings associated with other factors, e.g., sampling time, cultivar, or organ, are highlighted in blue. Metabolites were grouped according to functional categories, indicated in capital letters. The complete names of secondary metabolites can be retrieved from Supplementary Table 3.

Third, an integrated PCA was used to generate an overview of the variance in the whole data matrix, including stress and recovery responses (Figure 6A). MANOVA results were consistent with PCAs. Organ-specific differences in metabolic products dominated the variances, with roots clustering apart from leaves without overlap on PC1, which represented 68.53% of the variation between groups. Metabolites differed more between treatments than between cultivars, although for secondary metabolites, Fradel samples clustered apart from Pinhel samples. PC2 (13.44% of total variance) was associated with metabolic responses to stress/rewatering, with isoleucine, proline, quercetin 3-*O*-6″-malonylglycoside, quercetin, galactinol, quercetin 3-*O*-diglucoside-4-*O*-glucoside, and phenylalanine the main contributors with positive coordinates, and unknown7, glycerate, glucose, and kaempferol 3-*O*-diglycoside the main contributors with negative coordinates (Figure 6B), as indicated by loading values (Supplementary Table 6). The loading plots of each class of compound separately unequivocally revealed galactinol, proline, glycerate (PC2−), and quercetin 3-*O*-6″-malonylglycoside, quercetin (PC2+), kaempferol 3-*O*-diglycoside, and unknown7 (PC2−) as the primary and secondary metabolites, respectively, contributing to the separation between watering treatments (Figure 6B).

**Figure 7:**
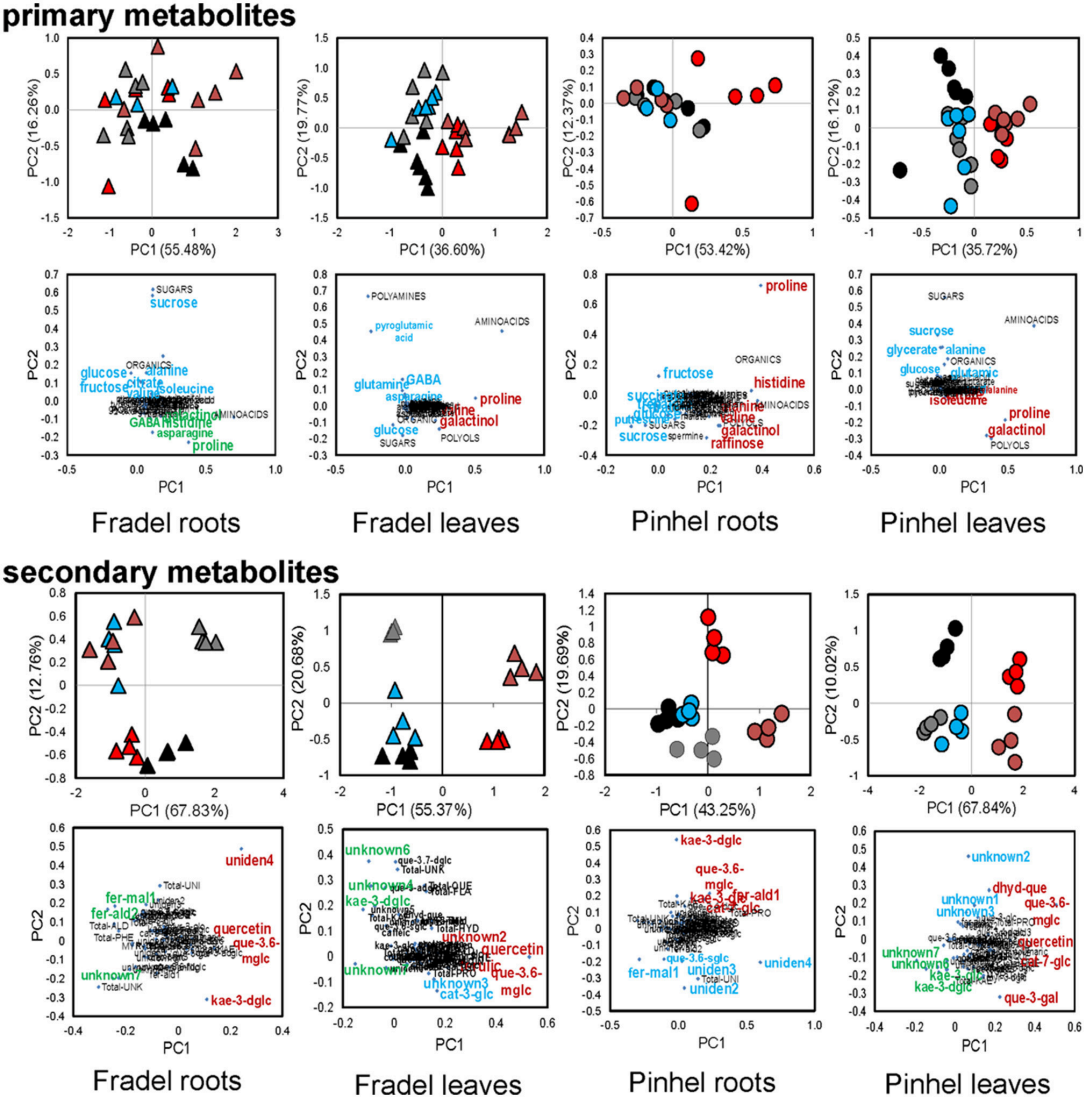
**Probabilistic PCA of 41 primary, 35 secondary, and 12 unidentified metabolites detected in cowpea under water deficit and recovery: levels in Fradel (triangles) and Pinhel (circles) roots and leaves that were measured under the following conditions: well watered (black and gray for 6 and 12 days of stress, respectively), drought stressed (red and brown for 6 and 12 days of stress, respectively), and rewatered (blue for plants stressed for 6 days and then rewatered for 6 days)**. Values representing up to six biological replicates were square root-transformed with pareto scaling. For each group of compounds, the upper plots represent PCA scores of the first two principal components, and lower plots represent the loadings for each compound. The highest positive and negative loadings (Supporting Information Supplementary Table S6) associated to metabolic responses to stress/rewatering are tagged in red and green, respectively. Loadings associated with other factors (e.g., sampling time) are highlighted in blue. Metabolites were grouped according to functional categories, indicated in capital letters. For full compound names, please refer to Supporting Information Supplementary Table S3.

**Figure 8:**
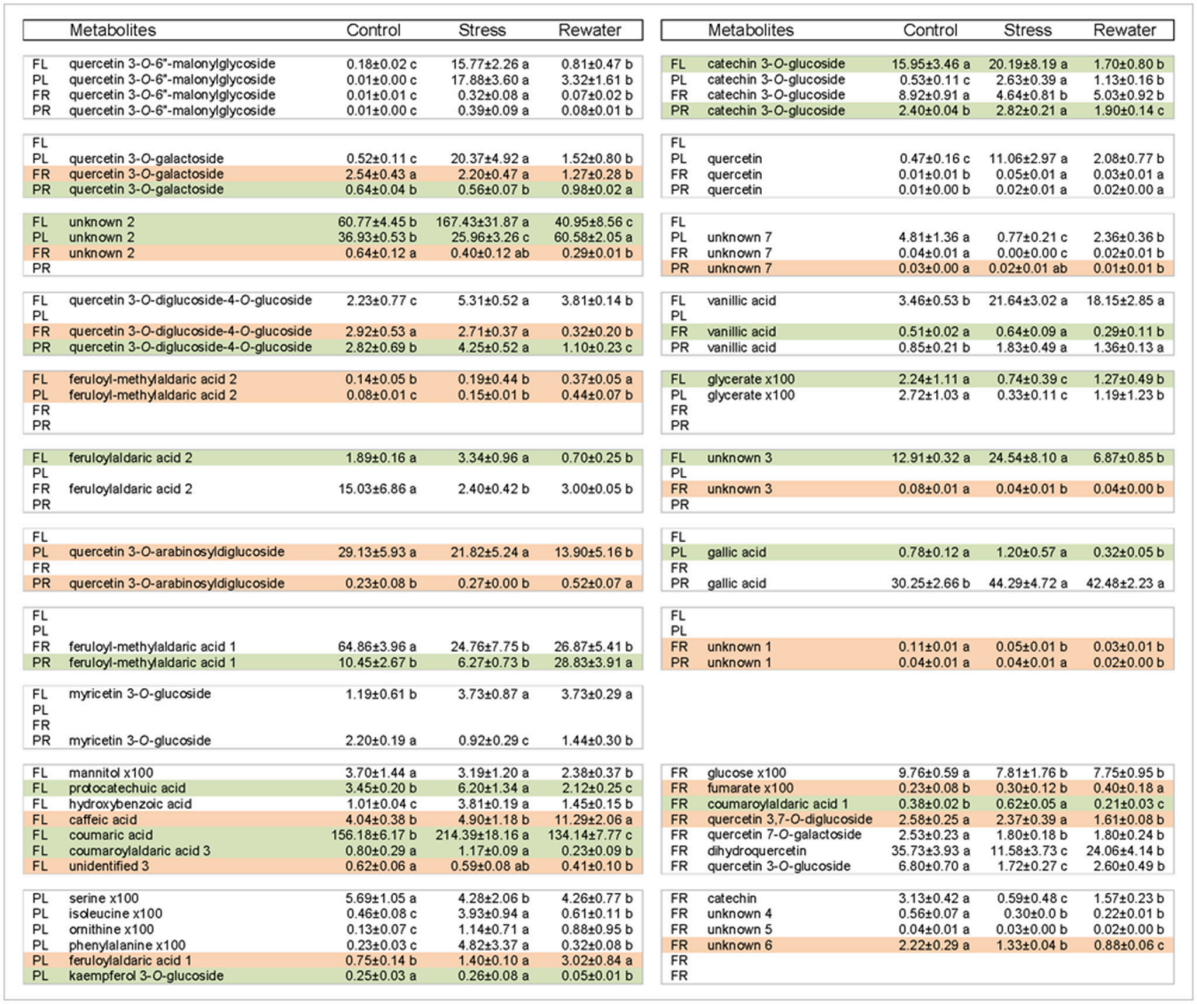
**Recovery performance of cowpea metabolites upon rewatering 12 days after drought stress**. Shown are metabolites whose levels differed between well-watered control and rewatered plants. The letters indicate cultivars: PL for Pinhel leaves, PR for Pinhel roots, FL for Fradel leaves, and FR for Fradel roots. White, compounds that did not fully recover; Green, compounds that overcorrected compared to the control; Red, compounds that continuously changed. Normalized relative values for primary metabolites are multiplied by 100 for easier comparison. Different letters inside the rows denote statistical differences between means (Tukey's test; *P* < 0.05).

Fourth, relationships between metabolites in roots and leaves were explored separately for each cultivar in additional PCAs (Figure 7). The PCAs indicated that nearly all control replicates were well-separated from drought-stressed replicates. In agreement with MANCOVA, progressive water deficit led to increased separation between treatments in the PCAs. With the exception of primary metabolites in Fradel roots, the drought treatment (PC1) was the main source of variance in the data. The top positive and negative loadings of PCs, distinguishing watering treatments for each cultivar and organ, corresponded to compounds that were previously selected using *t*-tests (Figure 3), with the addition of alanine, valine, catechin 7-*O*-glucoside and kaempferol 3-*O*-glucoside in Pinhel roots, and γ-aminobutyrate, galactinol, and unidentified4 in Fradel roots (Supplementary Table 6). The main compounds contributing to the dispersion of samples on the PCs and common to leaves and roots were proline, galactinol, quercetin 3-*O*-6″-malonylglycoside, and kaempferol 3-*O*-diglycoside. Rewatered replicates usually formed a distinct cluster near control replicates (except those corresponding to secondary metabolites in Fradel roots), indicating that plants reverted to their initial metabolic configuration (Figure 7). Indeed, Figure 4 showed that only 57% of Fradel root metabolites returned to normal status, whereas the percentage was over 75% for the other organs. Primary metabolites recovered more effectively, with only mannitol, glycerate (Fradel leaves), glucose, fumarate (Fradel roots), serine, isoleucine, ornithine, phenylalanine, and glycerate (Pinhel leaves) lagging behind (Figure 8). Besides quercetin and quercetin 3-*O*-6″-malonylglycoside, which were only partially restored after drought relief in all organs (suggesting they may be important during recovery), other compounds showed no biologically relevant pattern.

Fifth, treatments were grouped by similarities among variables using HCA (Figure 5A). The groups clustered according to watering regime, with a clear separation between stress and rewatering treatments, but also between the responses of leaves and roots to drought. However, in Fradel roots, recovery performance differed. For cultivars, the response was more variable and no clear separation was observed. Overall, quercetin 3-*O*-6″-malonylglycoside, kaempferol 3-*O*-diglycoside, quercetin, unknown7, galactinol, and proline (Figure 5B) responded the most to drought, as indicated by all statistical analyses. Five of these compounds grouped in the upper cluster of the hierarchy in Figure 5A, suggesting a coordinated response to drought.

### Drought responses in leaves and roots

**Figure 9:**
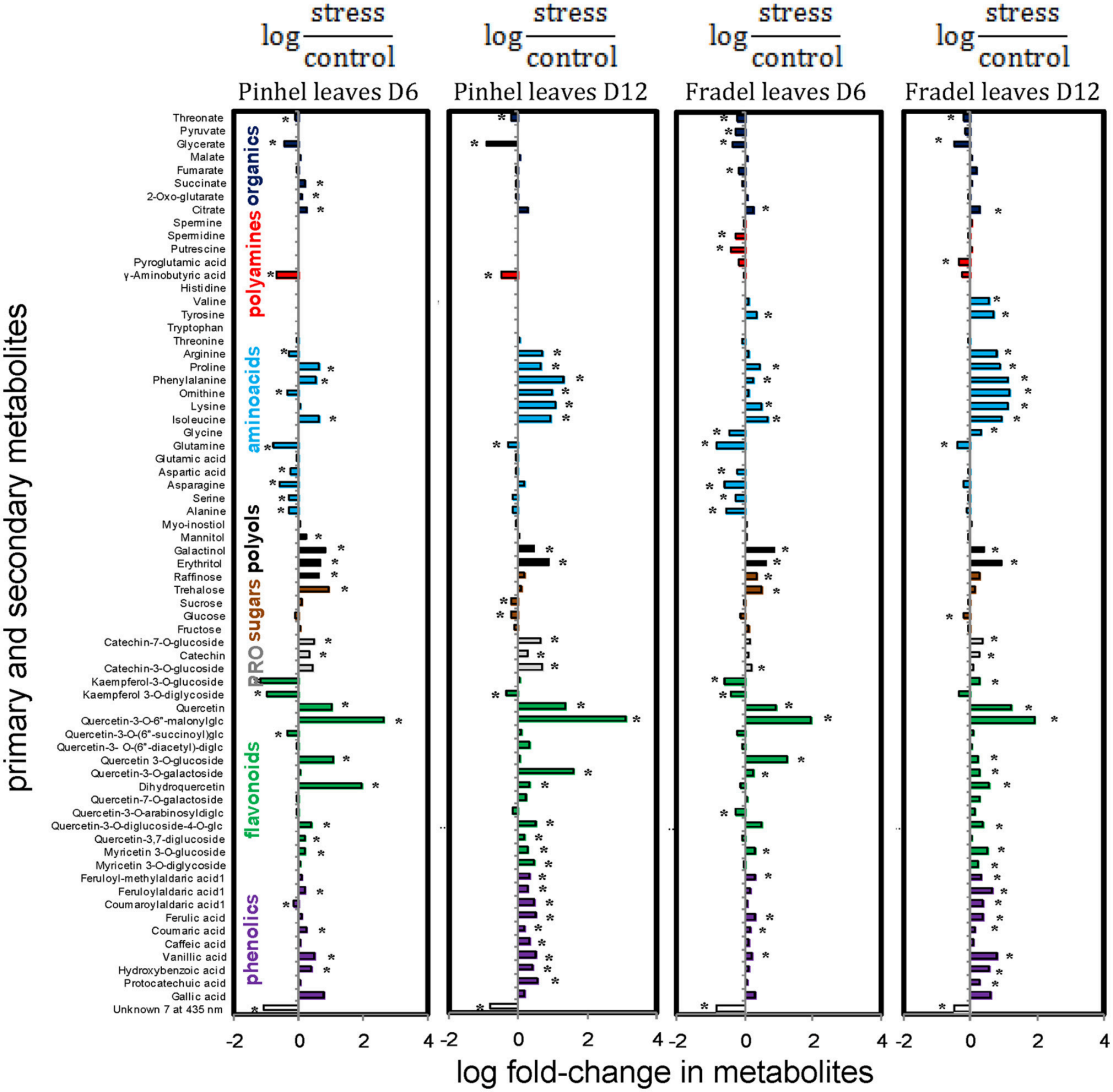
**Effects of drought stress on cowpea leaf metabolites**. Data are presented as averaged logarithmic fold-change ratios of metabolite levels in drought-stressed and control plants. Unidentified and undetected metabolites (with the exception of Unknown 7) are omitted for visual clarity. PRO = proanthocyanidins. Measurements were taken on day 6 (D6) or 12 (D12) of water stress (*n* = 4–6). Responses are represented by bars that indicate increased levels in drought-stressed plants (positive values) or decreased levels (negative values). Asterisks indicate values that were determined to be statistically different using *t*-tests (*P* < 0.05).

**Figure 10:**
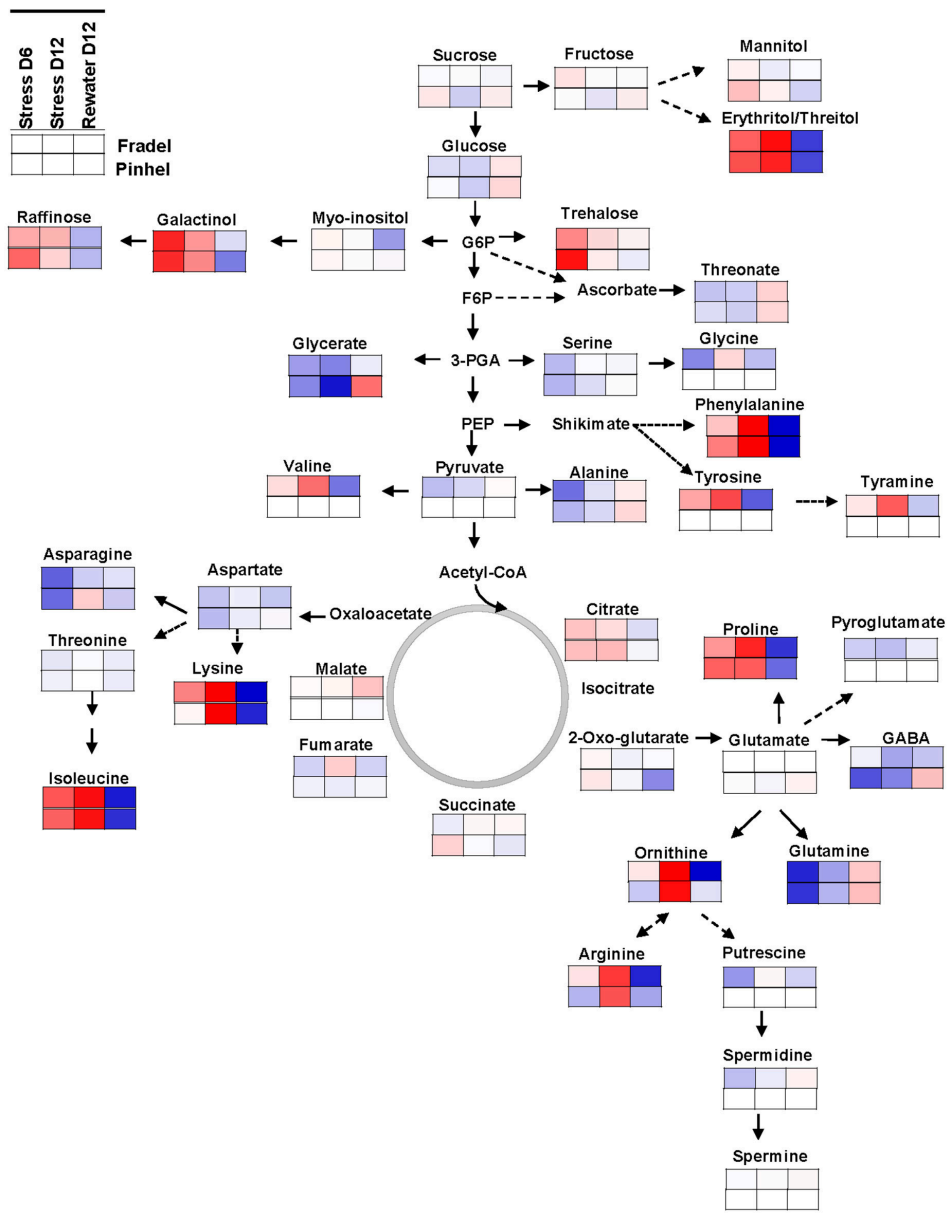
**Mapping of leaf metabolites in representative pathways**. Metabolites are visualized using the averaged log fold-change ratios (*n* = 4 to 6) between drought-stressed and well-watered plants harvested at day 6 and 12, and between rewatered and drought-stressed plants harvested at day 12. Fradel = top row cells; Pinhel = bottom row cells. Red and blue indicate increased and decreased levels, respectively.

Interestingly, compounds that increased or decreased in response to drought in the leaves were similar for Fradel and Pinhel. The following sustained trends were observed: (i) half of the organic acids increased while the others decreased; (ii) kaempferol and amino acid derivatives decreased; (iii) polyols, phenolic acids, unidentified phenolics, myricetin, quercetin, and catechin derivatives all increased; (iv) alanine, serine, asparagine, aspartate, glutamate, glutamine, glycine, and threonine decreased while the remaining amino acids increased; (v) raffinose and trehalose increased while sucrose, glucose, and fructose decreased; and (vi) half of the unknown compounds increased while the others decreased (Figures 9, 10).

**Figure 11:**
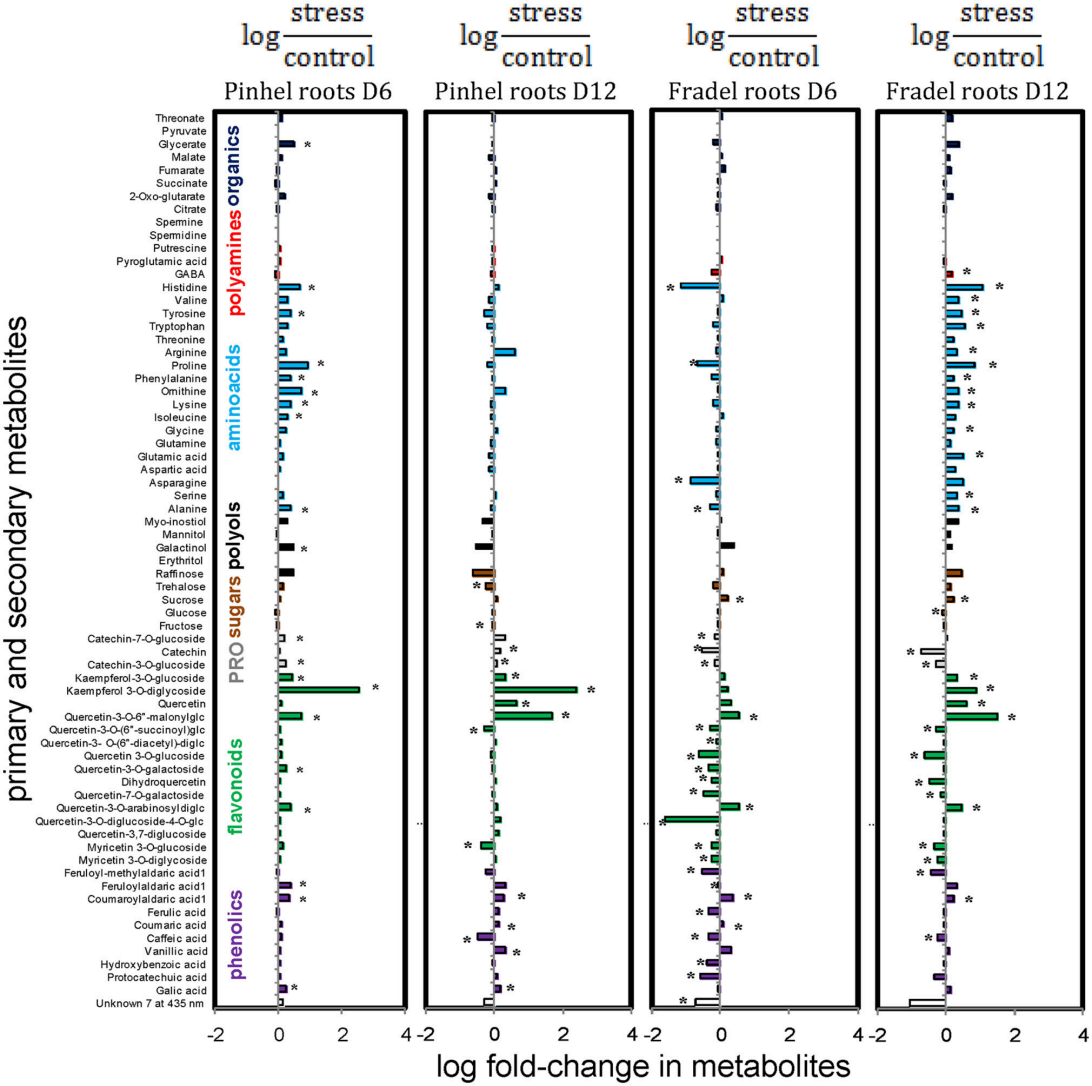
**Effects of drought stress on cowpea root metabolites**. Data are presented as averaged logarithmic fold-change ratios of metabolite levels in drought-stressed and control plants. Unidentified and undetected metabolites (with the exception of unknown7) are omitted for visual clarity. PRO = proanthocyanidins. Measurements were taken on day 6 (D6) or 12 (D12) of water stress (*n* = 4–6). Negative values represent reduced and positive values increased metabolite content in the stressed plants, with asterisks indicating statistical differences (*t*-test; *P* < 0.05).

In the roots, however, metabolites of Fradel and Pinhel generally shifted in opposite directions (Figure 11; Supplementary Figure 4). Pinhel roots initially accumulated both primary and secondary metabolites, but as drought stress continued, primary metabolites returned to control levels or decreased; although several secondary metabolites continued to increase, others were reduced. Meanwhile, in Fradel roots, primary and secondary metabolites initially decreased in response to drought stress; but as stress was prolonged, primary metabolites increased and several secondary metabolites recovered somewhat to levels that were still lower than the control. In general, metabolites belonging to same biochemical group changed in concert in roots but not in leaves. Another marked difference was with kaempferol 3-*O*-diglycoside, which decreased in the leaves in response to water deficit, but increased in the roots.

### Correlation between metabolites and grain yield

**Figure 12:**
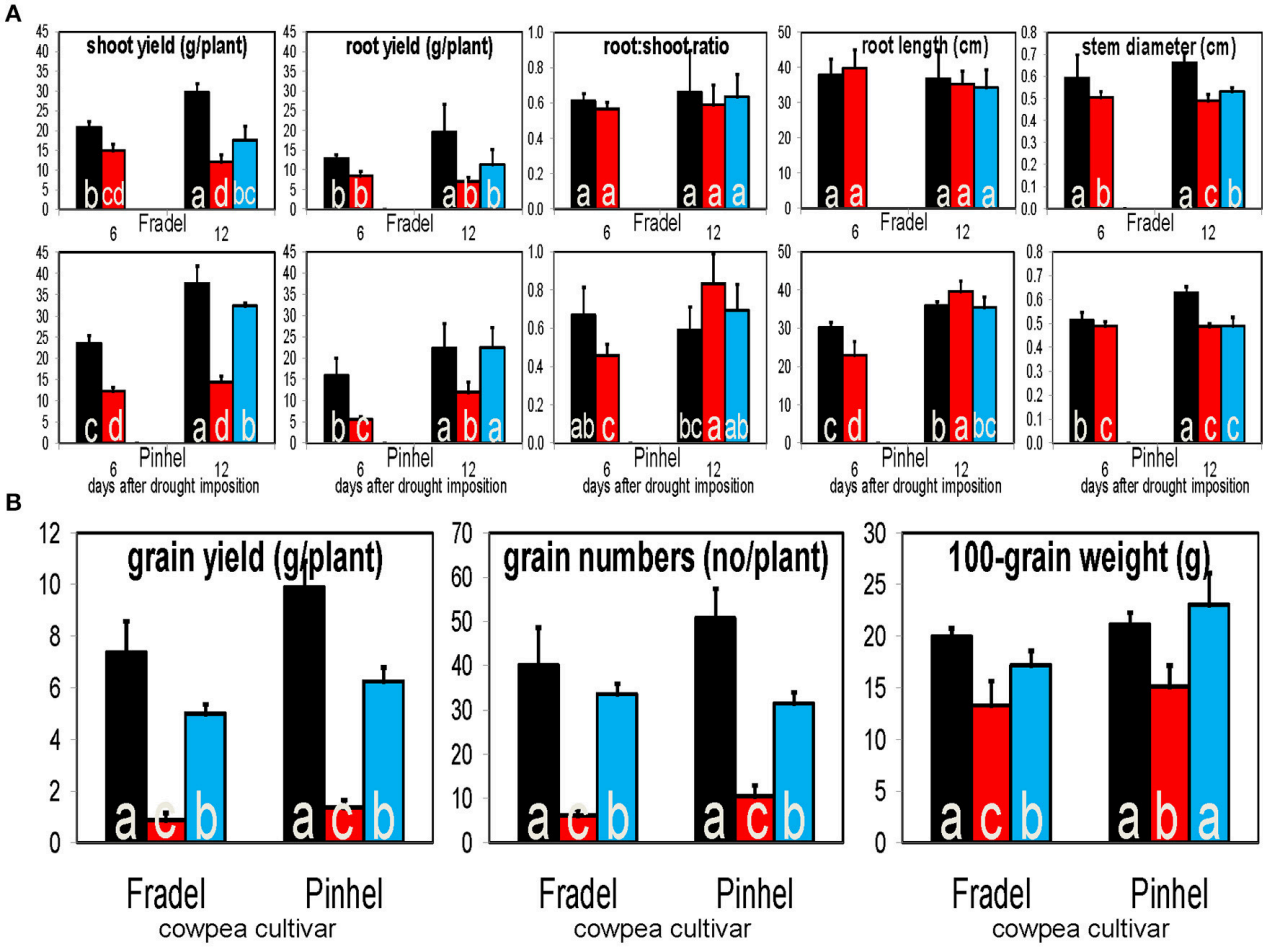
**Responses of cowpea fresh biomass, root length, and stem diameter (A)**, and grain yield and yield-related parameters **(B)**, to drought stress and rewatering. Bars indicate well-watered controls (black), drought stress for 6 or 12 days (red), and rewatering after 6 days of drought stress (blue; *n* = 6; error bars = SD). Different letters inside the columns for each cultivar denote statistical differences (Tukey's test; *P* < 0.05).

**Figure 13:**
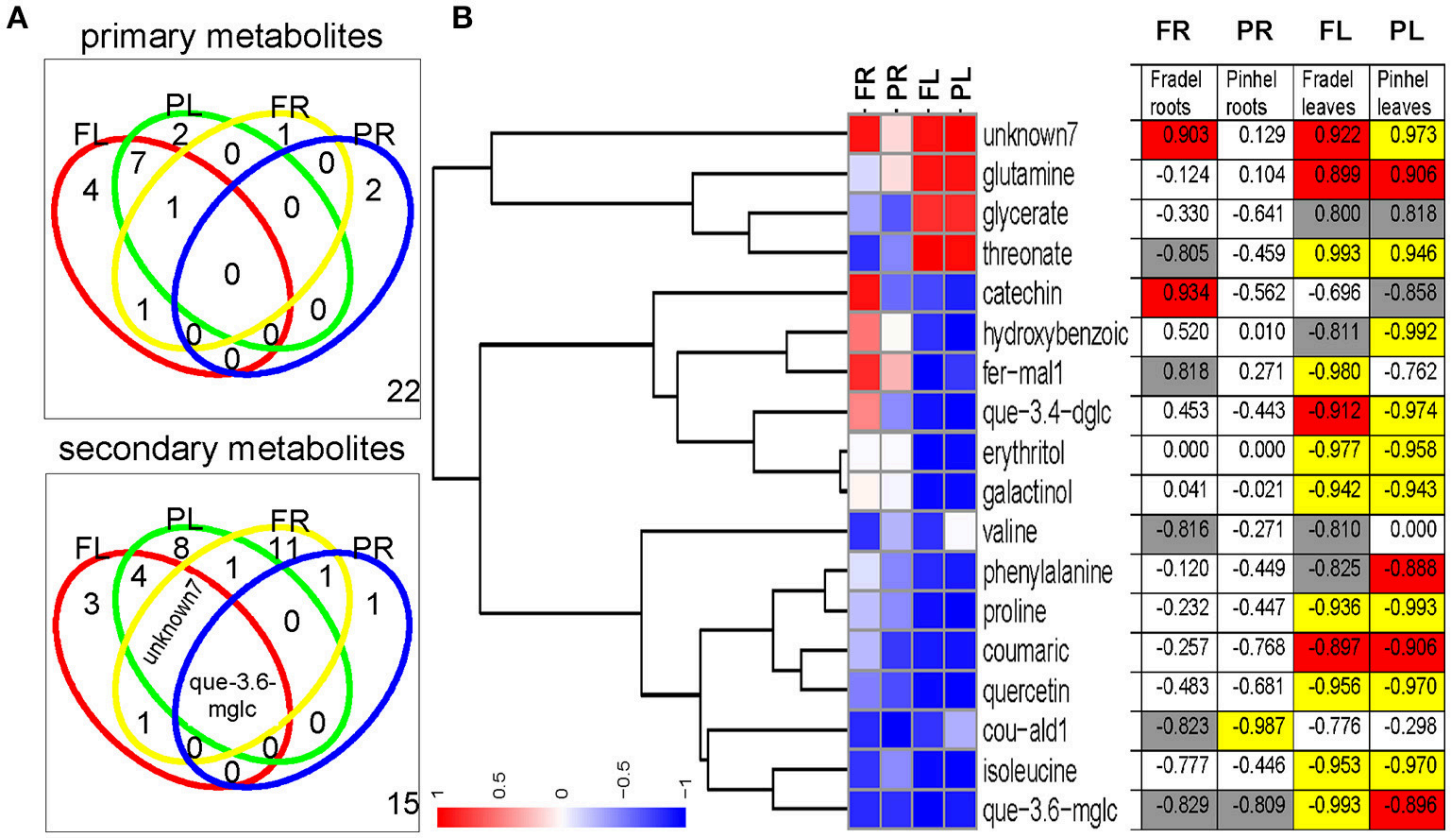
**Identification of metabolites influencing grain yield under drought stress with Pearson correlation analysis of log_**2**_-transformed means (full data provided in Supplementary Table 7). (A)** Venn diagrams indicating the number of common and specific metabolites correlated with grain yield for each cowpea cultivar. The threshold cut-off value for the metabolite–grain yield *r* was set at 0.800 and the number in the lower right quadrant represents metabolites that did not meet that criterion. **(B)** Complete linkage tree representation of metabolites correlated with grain yield in at least two of the comparisons, using correlation distance. Significant *r* values are highlighted in yellow (*P* < 0.01), red (*P* < 0.05), and gray (*P* < 0.10). fer-mal1, feruloyl-methylaldaric acid 1; que-3.4-dglc, quercetin 3-*O*-diglucoside-4-*O*-glucoside; cou-ald1, coumaroylaldaric acid 1; que-3.6-mglc, quercetin 3-*O*-6″-malonylglycoside.

Water stress negatively affected all measured yield parameters, even after rewatering (Figure 12B). Correlations between metabolite levels and grain yield under all growth conditions showed that 18 primary and 32 secondary metabolites were correlated with grain yield with |*r*| > 0.800 in at least one of the four organ-by-cultivar comparisons (Figure 13A; Supplementary Table 7). Compounds (*n* = 18) present in at least two of the comparisons were further analyzed using HCA, which showed strong correlations (|*r*| > 0.990) between yield and quercetin 3-*O*-6″-malonylglycoside, proline, hydroxybenzoate, and threonate (Figure 13B).

### Effect of drought and rewatering on water relations and elemental solutes

**Figure 14:**
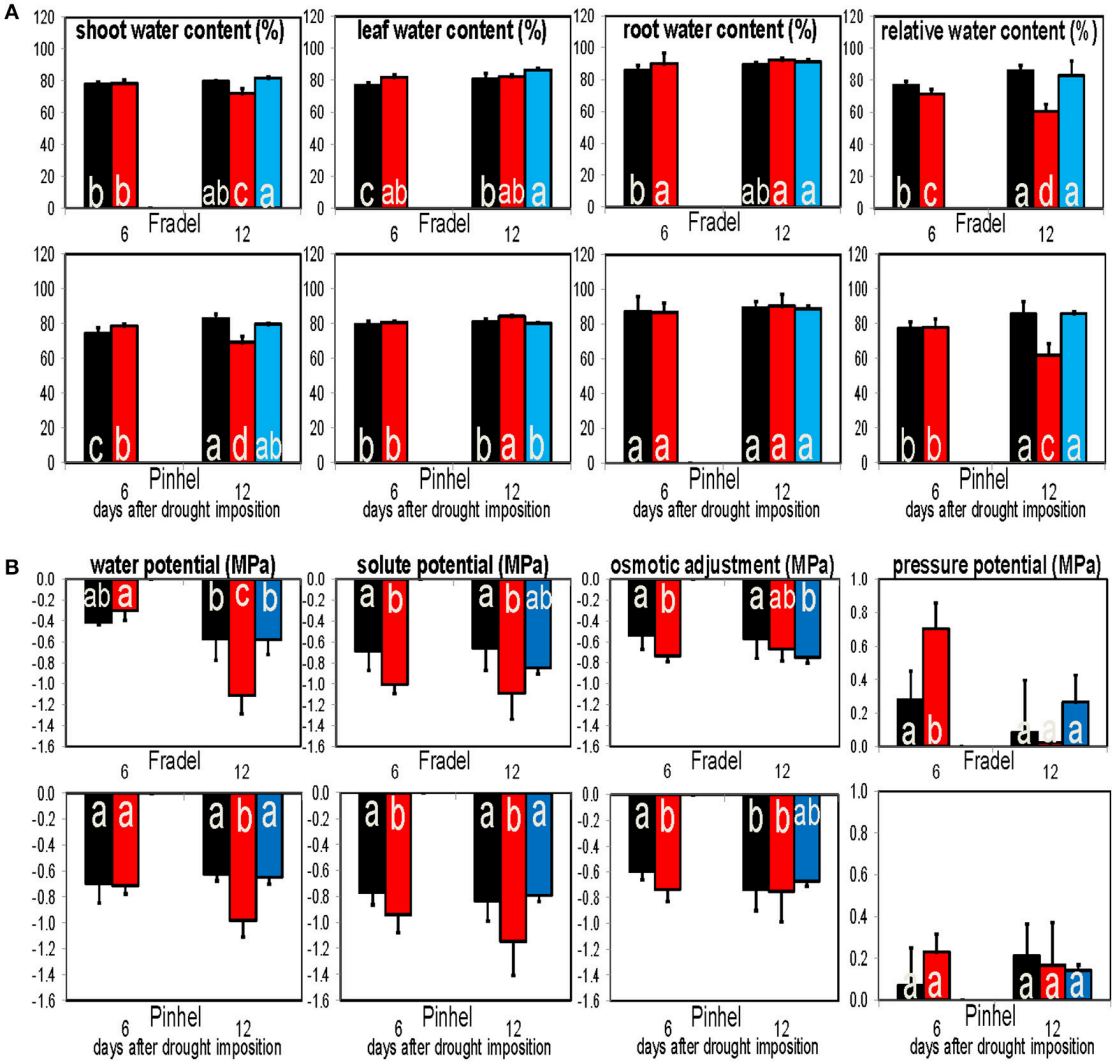
**Responses of water content (A)** and water potential components **(B)** to drought stress and rewatering. Pressure potential, water potential—solute potential; Osmotic adjustment, osmotic potential values corrected to full turgor by multiplying with relative water contents. Bars indicate the well-watered controls (black), plants drought-stressed for 6 or 12 days (red), and rewatered plants after 6 days of drought stress (blue; *n* = 6; error bars = SD). Different letters inside the columns for each cultivar denote statistical differences (Tukey's test; *P* < 0.05).

The leaf and root water content of drought-stressed plants were similar or higher (*P* < 0.05) than those of control plants at every harvest. The shoot water content was also similar or higher at D6 and decreased by D12 (Figure 14A). Relative water content correlated positively with water potential; for the two parameters, differences between treatments were more evident at D12, with decreased levels in stressed plants. At D6, decline of osmotic potential at full turgor occurred in both cultivars, indicating the development of a foliar osmotic adjustment process in response to water stress. The degree of adjustment was 0.20 MPa in Fradel, and 0.14 MPa in Pinhel. However, no significant differences in adjustment were apparent between well-watered and drought-stressed leaves at D12 (Figure 14B).

**Figure 15:**
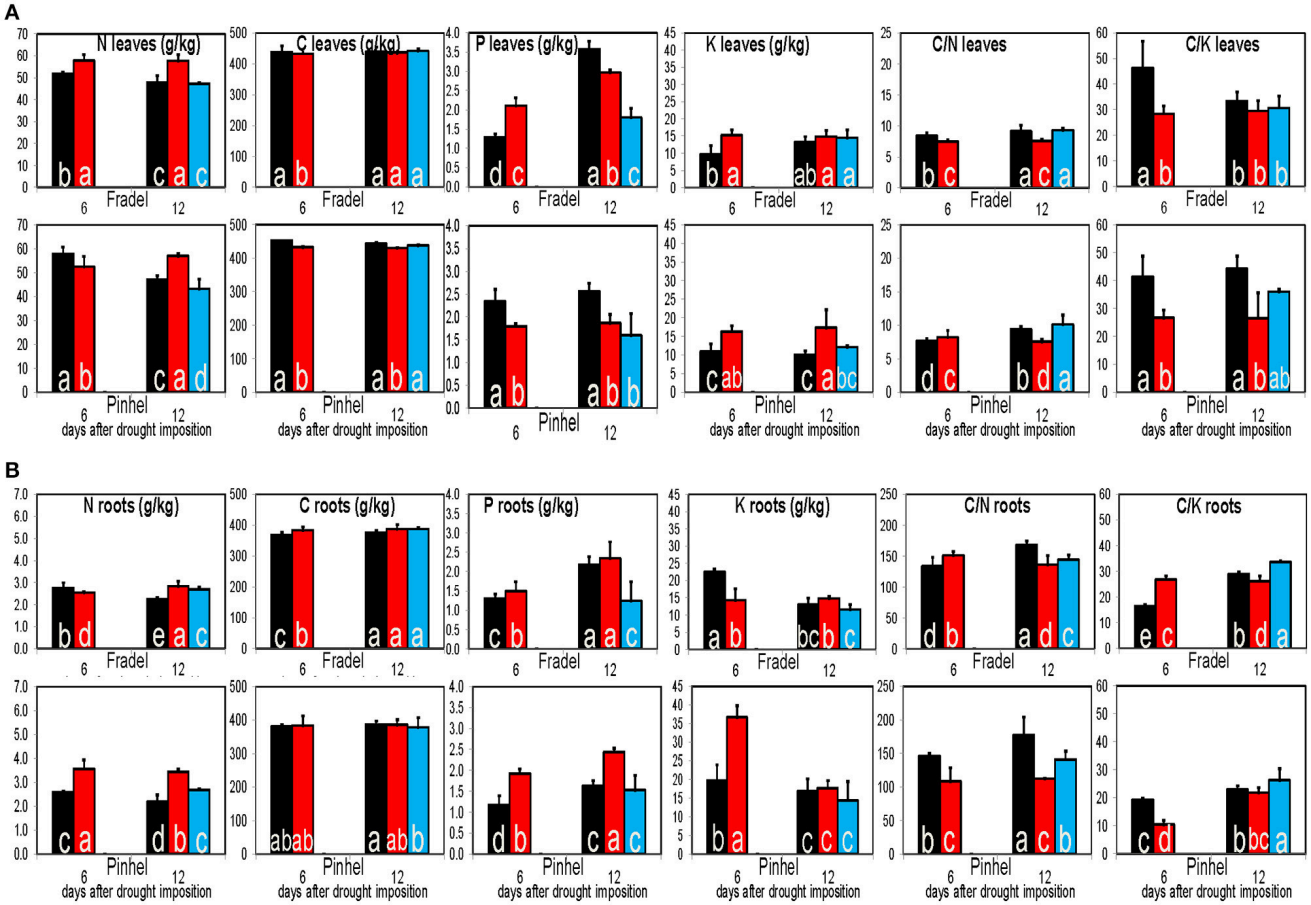
**Elemental solute levels in leaves (A)** and roots **(B)** of cowpea in response to drought stress and rewatering. Bars indicate the well-watered controls (black), plants drought-stressed for 6 or 12 days (red), and rewatered plants after 6 days of drought stress (blue; *n* = 3–6; error bars = SD). Different letters inside the columns for each cultivar denote statistical differences (Tukey's test; *P* < 0.05).

In general, drought decreased the C/N ratio except in Pinhel leaves and Fradel roots at D6 (Figure 15A). Drought-stressed plants usually had higher K content (resulting in a low C/K ratio), whereas P content decreased and increased in leaves and roots, respectively (Figure 15B). A PCA that included data from the elemental analyses clearly identified K and C/K as important factors in drought response (Supplementary Figure 5). With few exceptions, values returned to control levels after rewatering.

## Discussion

Plants tolerate drought by producing organic solutes, which act as compatible osmolytes that accumulate in the cytosol for osmotic adjustment and turgor maintenace, or osmoprotectants that stabilize cellular constituents. This study looked at identifying qualities of osmotic adjustment and osmoprotection in plants, using cowpea as the model species. Cowpea is considered a drought-resistant crop, but drought still constrains its productivity. Delayed leaf senescence, stem greenness, and deep rooting have been identified as important traits for enhancing cowpea grain yield under water stress (Muchero et al., 2008). However, phenotype-based selection has been relatively slow, mainly owing to the unpredictability of drought onset and considerable environmental effects on phenotypic expression (Hamidou et al., 2007; Hall, 2012). Recent efforts have concentrated on the use of DNA-based markers derived from quantitative trait loci (Agbicodo et al., 2009; Shui et al., 2013). It is expected that drought tolerance may also be conferred by engineering cowpea stress responses via metabolite-based markers (Khan et al., 2015). Cowpea has a remarkable ability to withstand drought conditions by limiting water loss. Although drought avoidance strategies, such as stomatal closure and paraheliotropism, have well been described in cowpea, the possibility of moisture conservation through osmolyte accumulation has not been adequately examined.

In this study, cowpea grown in greenhouse was subjected to drought for 6 or 12 days and rewatered for 6 days after 6 days of stress (Figure 1). In the experimental region, cowpea growing season is characterized by abrupt rainfall. In 2016, for example, one rainfall event of 13.2 mm occurred in mid-June, 1 month after cowpea sowing (Supplementary Figure 1). That rainfall was sufficient to mimic greenhouse rewatering conditions for up to 2 weeks, based on soil data (not shown). Therefore, the adopted design simulated ecologically realistic stress and provided a valuable opportunity to study cowpea adaptation to new watering regimes.

### Photosynthetic data suggest a better resistance of pinhel than fradel to drought

Harvest dates were scheduled based on the loss and recovery of *A*, which occurred during the vegetative and reproductive stages, respectively. Cowpea responded to water deficit by showing a strong relationship between *A* and field capacity. Decreased photosynthesis is usually linked to limitations to CO_2_ assimilation imposed by stomatal closure, but can also result from impaired mesophyll conductance and/or biochemical and photochemical constraints (Pinheiro and Chaves, 2011). Increased Ci/Ca (intercellular CO_2_/ambient CO_2_) and reduced *A*/g_*s*_ (intrinsic water use efficiency) were observed by D6 (Figure 2A), which indicated non-stomatal photosynthesis limitation. These changes coincided with an increase in NPQ (non-photochemical quenching coefficient; Figure 2B), suggesting prevention of photo-oxidative damage of the photosynthetic apparatus. Moreover, the fast recovery after irrigation suggests that other non-assimilatory processes had a role; the Mehler reaction and/or photorespiration, for example, may be enhanced to partially dissipate excess excitation energy or scavenge any reactive oxygen species produced (Pinheiro and Chaves, 2011; Sánchez-Martín et al., 2015). Therefore, photoinhibition was unlikely a major driver of drought stress in the cultivars. Pinhel, however, exhibited a finer modulation of the photosynthetic process than Fradel.

### Drought stress progressively affects the cowpea metabolome

An important objective of this work was to identify metabolite markers for drought adaptation in cowpea. The approach used centered on the hypothesis that comparatively better survival and grain yield under drought and recovery must be reflected in developmental-, organ-, and cultivar-specific similarities in metabolic responses. Levels of 88 metabolites were measured during the vegetative and reproductive stages to assess metabolic acclimation to drought stress. Data showed that changes in the metabolome were more severe with extended periods of stress, irrespective of developmental stage (Figure 4). Therefore, flowering did not seem to alter metabolic responses to drought as seen with rice (Li et al., 2015; Raorane et al., 2015), and a progressive metabolic acclimation was indicated for the two cultivars. A unified response was evident from all statistical analyses (Student *t*-test, MANOVA, PCA), and quercetin 3-*O*-6″-malonylglycoside, kaempferol 3-*O*-diglycoside, quercetin, unknown7, galactinol, and proline were identified as having the most significant responses to drought stress (Figure 5B), assuming that fold-changes under stress, not basal levels, indicate resistance markers (Johnson et al., 2015). These conserved metabolic responses likely reflect the basic lynchpin metabolic acclimation of cowpea to drought stress.

### Metabolic responses to drought are similar in leaves, but not in roots

The present study also examined whether roots or leaves would yield more metabolic information regarding drought adaptation. Metabolic responses to drought in leaves were similar during vegetative and reproductive growth (Figure 9). In roots, however, cultivar-specific metabolic responses were observed and were modulated by drought intensity, duration, and/or rate of progression (Figure 11). The changes in metabolic responses observed in roots could not be explained by differential growth inhibition, as suggested by Sanchez et al. (2012); genetic variation between cultivars seems more plausible. As cowpea responds to drought, metabolic pathways may be regulated in dissimilar orders before an optimal balance is established. However, harvest dates in this study were too distant to establish the timing of these changes. Moreover, changes in root metabolites could be part of a signaling network. The hormone abscisic acid acts as a dominant root-to-shoot signal under water deficit (Costa et al., 2011; Kwasniewski et al., 2016). Other signals have not been fully identified, and the interplay of hormones with sugars and redox signals is the focus of current research (Pinheiro and Chaves, 2011; Blum, 2017). The root metabolome as a whole, rather than a specific compound, could be acting as a signal transducer, thus modulating photosynthate investment in different parts of the plant. In summary, the data showed that both leaves and roots could be used to record changes in water conditions; however, leaves are probably more suitable for identifying water stress, while roots may exhibit distinct metabolic changes that indicate specific drought resistance mechanisms.

### Changes in grain yield reflect changes in metabolic signatures in response to drought

Potential stress markers are often identified as amenable to biotechnological exploitation based on the magnitude of their change. However, high levels of specific compounds that do not provide concordant yield increases are irrelevant (Hill et al., 2013). Therefore, the metabolic responses obtained in this study were placed in an agricultural perspective by combining them with yield data (Figure 13). Owing to their responsiveness, as highlighted by fold-changes and metabolite-yield correlations, three compounds were chosen as promising markers for yield performance under drought stress, namely proline, galactinol, and quercetin 3-*O*-6″-malonylglycoside. Although quercetin and unknown7 were significantly correlated with grain yield, they were not very responsive to drought stress in the roots. Curiously, kaempferol 3-*O*-diglycoside, which was consistent between cultivars and organs, failed to correlate with grain yield; it may respond regardless of the plant's ultimate ability to survive under stress.

### Cowpea metabolic regulation under drought is compatible with osmoprotection

To determine whether the observed metabolic responses indicated tolerance mechanisms, such as osmotic adjustment or cell wall extensibility, changes in osmosis with progressing drought stress were evaluated using osmotic potential, C/N ratio, and K data (Lugan et al., 2010; Gargallo-Garriga et al., 2015; Blum, 2017).

An “osmotic adjustment” indicates that solutes in plant tissues (osmotic potential) have accumulated at least as much as water potential or relative water content has been reduced (Blum, 2017); Fradel and Pinhel demonstrated such an adjustment, but only during the first days of stress (Figure 14B). The small and transient adjustment observed was unlikely the result of organic solute accumulation in the cells, given the cumulative effect of stress on the metabolome. Under water stress, N generally accumulated to comparable levels in both cultivars at the expense of C (Figure 15), in line with previous studies in grapevine (Hochberg et al., 2013) and rice (Raorane et al., 2015). The reduction in photosynthetic capacity during drought is thought to contribute to the shrinking of the total C pool (Jorge et al., 2015), which could explain the negative C balance observed. Enzymatic and molecular evidence argue for a negative effect of water deficit on N assimilation (Raorane et al., 2015). Thus, increased N can better be explained by increased catabolism of stored amino acids and protein pools. Cowpea evidently regulated its metabolism to balance the production of N- and C-containing metabolites, as indicated by increases and decreases in an equal number of primary metabolites in the leaves (Figure 9), which suggests that the total solute content remained constant.

Osmotic effects are controlled not only by organic compounds but also by inorganic solutes. K^+^ has a particularly well-known role in plant water balance (Gargallo-Garriga et al., 2015). As shown in Figure 15, K levels were generally much higher in drought-stressed plants, which could better account for the increase in cell osmolality of drought-stressed leaves. Therefore, K-based osmotic adjustment during the early stages of stress may constitute an important and energetically efficient adaptive feature in cowpea, but is insufficient in sustaining turgor under more severe stress conditions.

Overall, the data indicate that metabolic changes in cowpea are closely regulated and do not occur in isolation. Although these changes might not make a significant contribution to osmotic adjustment, they could still ensure appropriate homeostatic maintenance via non-osmotic roles, such as redox buffering, membrane stabilization, protein hydrotroping, radical scavenging, signaling, or N and C repository building (Sanchez et al., 2012; Johnson et al., 2015; Jorge et al., 2015; Blum, 2017). In the present study, this is indicated by the accumulation of several flavonoids with antioxidant activities (Nakabayashi et al., 2014; Simova-Stoilova et al., 2015) such as quercetin derivatives and proanthocyanidins with cell wall-stiffening functions (Hachibamba et al., 2013; Hochberg et al., 2013) such as catechin derivatives. The general emerging picture is that the cowpea metabolic configuration under water deficit is compatible with osmoprotective strategies, at least in the leaves, indicating that changes in proline, galactinol, and quercetin 3-*O*-6″-malonylglycoside levels are an integral part of an adaptive response rather than stress indicators. In the particular case of proline, the observed changes were unlikely a symptom of damage, given the high level of congruence between the responses to stress and rewatering and the differing responses in roots. Moreover, the accumulation of several other known compatible solutes (Hochberg et al., 2013; Meyer et al., 2014; Shelden et al., 2016) along with proline, such as phenylalanine, raffinose, and isoleucine, is unlikely to be coincidental. Thus, it is highly likely that the metabolite changes were functionally connected with an increase in resistance, and that their correlations with yield indicated beneficial effects.

### Metabolic changes indicate a possible role for the shikimate pathway in the protection of cellular functions

Finally, it was important to determine whether these compounds were under common genetic control via a specific pathway, which could facilitate breeding for drought tolerance. Regulation of most pathways was consistent in the leaves of both cultivars (Figure 10) but differed in the roots (Supplementary Figure 4). The leaf metabolic map indicated a major shift in C partitioning away from glycolysis/gluconeogenesis into the raffinose/stachyose (via galactinol) and the shikimate (via phenylalanine) pathways. There is an increasing body of evidence for the implication of raffinose family oligosaccharides (RFOs) in drought tolerance (Lugan et al., 2010; Hochberg et al., 2013; Li et al., 2015). Myo-inositol, for example, has recently been identified as the most promising metabolite marker for increased maize yield under drought stress (Obata et al., 2015). However, RFOs may primarily serve as transient C storage sources, with advantages in mobility over sucrose and starch (Obata et al., 2015). The accumulation of branched-chain amino acids (valine/leucine/isoleucine pathway) may also be associated with storage of substrates for key metabolic pathways. For all combinations of cultivars and organs, differences in the amounts of phenylalanine were strongly related to its downstream metabolism, i.e., accumulation of phenolics. Interestingly, enhanced drought tolerance in plants via overaccumulation of phenolics and other secondary metabolites has been documented (Nakabayashi et al., 2014; Corso et al., 2015). In the roots (Supplementary Figure 4), opposing regulation of glycolysis/gluconeogenesis was found, with a significant increase in sucrose levels, which suggested a possible shift in the localization of energy-intensive processes, such as biomass production, from leaves to roots (Raorane et al., 2015; Simova-Stoilova et al., 2015). In contrast, the arginine/proline pathway seemed to receive additional N inputs (Figure 10); the pathway was far more stimulated in Fradel than in Pinhel by D12, which could be related to the greater damage sustained during water stress in the former. The map also highlighted two other pathways with important roles, with decreased levels of metabolites of the glycine/serine/threonine pathway, suggesting faster consumption of these metabolites through enhanced photorespiration (Sánchez-Martín et al., 2015) as indicated by chlorophyll fluorescence data, and the alanine/aspartate/glutamate pathway, suggesting increased energy consumption from reserves (Hill et al., 2013).

Overall, the mechanisms by which cowpea modifies its metabolism to meet the demands of diverse resistance functions when exposed to water deficit appear to be determined by the interplay between the shikimate and arginine/proline pathways, giving rise to three drought-responsive metabolites, namely proline, galactinol, and quercetin 3-*O*-6″-malonylglycoside. Mapping chromosomal regions jointly associated with these pathways and investigating their co-localization with quantitative trait loci in a larger population may indicate promising candidate genes for breeding.

## Author contributions

The work was conceived and designed by HT, ER, and PG. PG (greenhouse work, secondary metabolites, water content, elemental solutes), JM (chlorophyll fluorescence), CC (gas exchange), TJ, CA (primary metabolites), and MO (osmotic adjustment) performed the experiments and acquired the data. All authors analyzed and interpreted the results. PG drafted the manuscript, which was critically revised by all authors.

## Funding

This work was funded by EUROLEGUME (Seventh Research Framework Programme of the European Union—FP7 research project 613781), and supported by Portuguese National Funds (FCT—Fundação para a Ciência e a Tecnologia) through the projects UID/AGR/04033/2013 and POCI-01-0145-FEDER-006958, postdoctoral fellowship SFRH/BPD/73302/2010 (PG), Investigator Programme IF/00376/2012/CP0165/CT0003 (CA), and PhD fellowship PD/BD/113475/2015 (TJ). CA further acknowledges support from ITQB-NOVA R&D GREEN-it (UID/Multi/04551/2013), and TJ the ITQB-NOVA International PhD Programme “Plants for Life” (PD/00035/2013).

### Conflict of interest statement

The authors declare that the research was conducted in the absence of any commercial or financial relationships that could be construed as a potential conflict of interest.

## Supplementary material

Supplementary Figure 1 **Weather records for 2013–2016**.

Supplementary Figure 2 **Gas exchange with shared and divergent responses**.

Supplementary Figure 3 **HPLC chromatograms of cowpea samples**.

Supplementary Figure 4 **Mapping of root metabolites in pathways**.

Supplementary Figure 5 **PCA with element and organic solutes**.

Supplementary Table 1 **Soil data**.

Supplementary Table 2 **GC-TOF-MS data**.

Supplementary Table 3 **LC-DAD data**.

Supplementary Table 4 **Gas exchange and chlorophyll fluorescence data**.

Supplementary Table 5 **MANOVA data**.

Supplementary Table 6 **PCA loadings**.

Supplementary Table 7 **Yield-metabolites correlations**.
