# Supplementary material for: Cowpea (Vigna unguiculata L. Walp.) Metabolomics: Osmoprotection as a Physiological Strategy for Drought Stress Resistance and Improved Yield
Source: Front Plant Sci. 2017 Apr 20;8:586. doi: 10.3389/fpls.2017.00586 (PMC5397532; doi:10.3389/fpls.2017.00586)
Supplement: Supplementary Figure 1 — Weather records for 2013–2016. [file Image1.PDF]

Metabolomic of Cowpea (*Vigna unguiculata* L. Walp.) Leaves and Roots: Osmoprotection as a Physiological Strategy for Drought Stress Resistance and Improved Yield

Piebiep Goufo, José Moutinho-Pereira, Tiago F. Jorge, Carlos M. Correia, Manuela R. Oliveira, Eduardo A.S. Rosa, Carla António, and Henrique Trindade

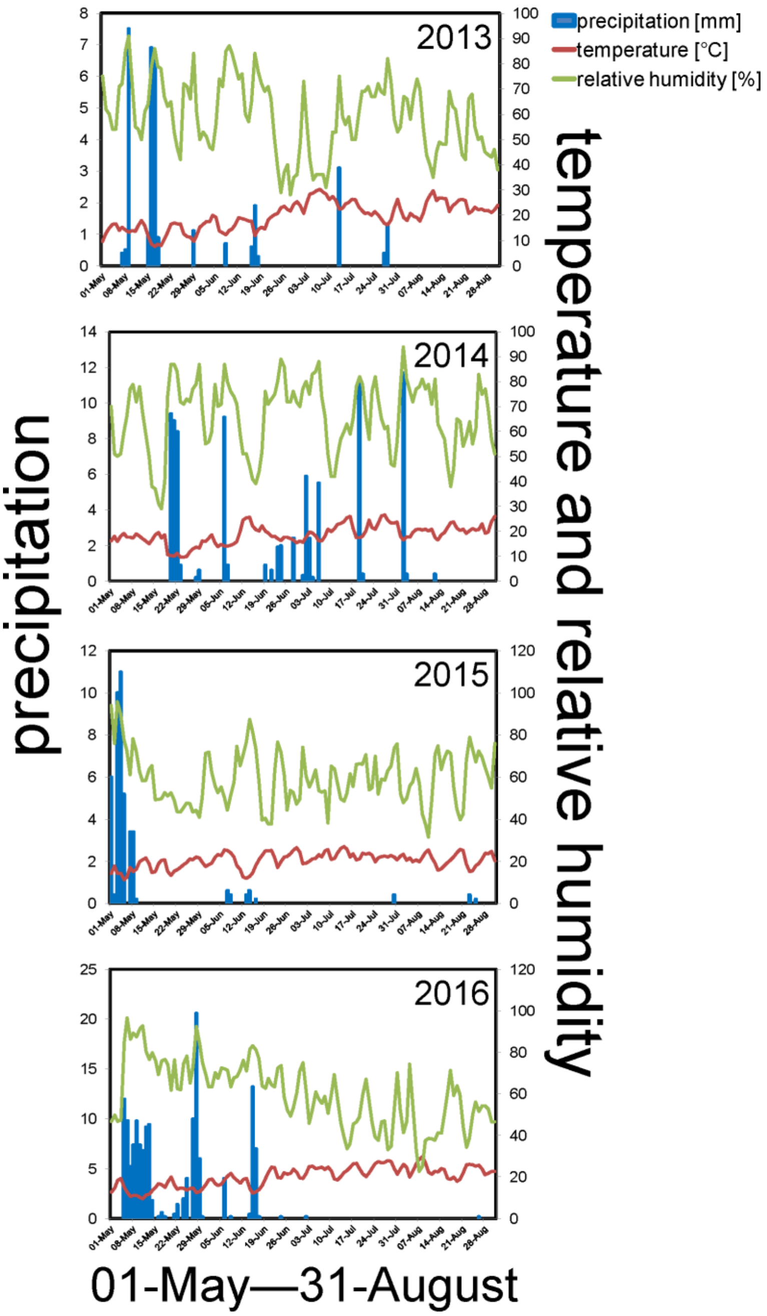

Supplementary Figure S1. The mean daily climatic parameters during 2013, 2014, 2015, and 2016 at the experimental farm of UTAD. Although the water deficit experiment was conducted in a greenhouse, drought stress and rewatering were scheduled to mimic rain events outside the greenhouse. Weather records in the greenhouse over the water deficit period are described in the text.

A

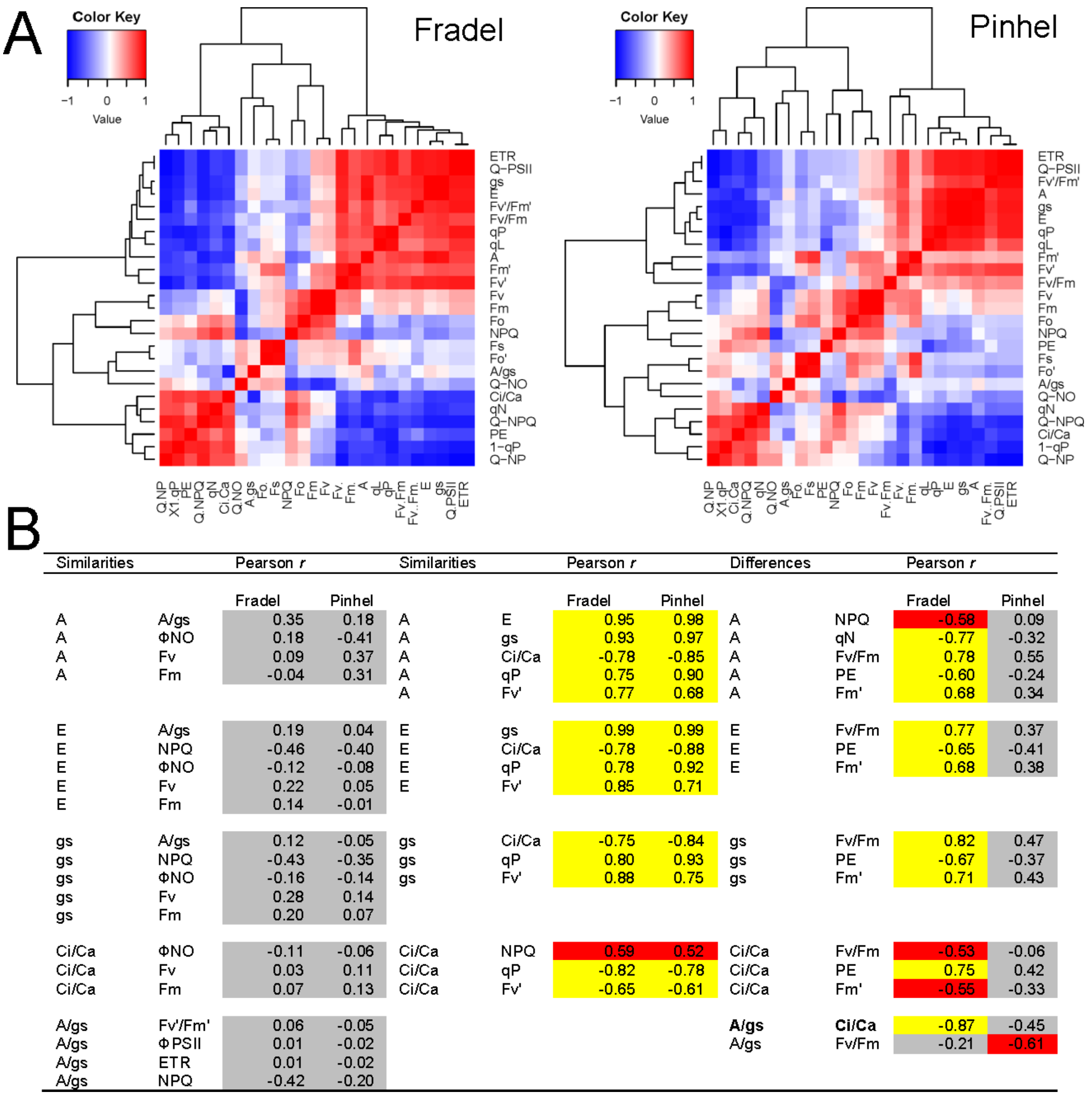

B

Similarities		Pearson *r*		Similarities		Pearson *r*		Differences		Pearson *r*	
		Fradel	Pinhel			Fradel	Pinhel			Fradel	Pinhel
A	A/gs	0.35	0.18	A	E	0.95	0.98	A	NPQ	-0.58	0.09
A	ΦNO	0.18	-0.41	A	gs	0.93	0.97	A	qN	-0.77	-0.32
A	Fv	0.09	0.37	A	Ci/Ca	-0.78	-0.85	A	Fv/Fm	0.78	0.55
A	Fm	-0.04	0.31	A	qP	0.75	0.90	A	PE	-0.60	-0.24
				A	Fv'	0.77	0.68	A	Fm'	0.68	0.34
E	A/gs	0.19	0.04	E	gs	0.99	0.99	E	Fv/Fm	0.77	0.37
E	NPQ	-0.46	-0.40	E	Ci/Ca	-0.78	-0.88	E	PE	-0.65	-0.41
E	ΦNO	-0.12	-0.08	E	qP	0.78	0.92	E	Fm'	0.68	0.38
E	Fv	0.22	0.05	E	Fv'	0.85	0.71				
E	Fm	0.14	-0.01								
gs	A/gs	0.12	-0.05	gs	Ci/Ca	-0.75	-0.84	gs	Fv/Fm	0.82	0.47
gs	NPQ	-0.43	-0.35	gs	qP	0.80	0.93	gs	PE	-0.67	-0.37
gs	ΦNO	-0.16	-0.14	gs	Fv'	0.88	0.75	gs	Fm'	0.71	0.43
gs	Fv	0.28	0.14								
gs	Fm	0.20	0.07								
Ci/Ca	ΦNO	-0.11	-0.06	Ci/Ca	NPQ	0.59	0.52	Ci/Ca	Fv/Fm	-0.53	-0.06
Ci/Ca	Fv	0.03	0.11	Ci/Ca	qP	-0.82	-0.78	Ci/Ca	PE	0.75	0.42
Ci/Ca	Fm	0.07	0.13	Ci/Ca	Fv'	-0.65	-0.61	Ci/Ca	Fm'	-0.55	-0.33
A/gs	Fv'/Fm'	0.06	-0.05					**A/gs**	**Ci/Ca**	-0.87	-0.45
A/gs	ΦPSII	0.01	-0.02					A/gs	Fv/Fm	-0.21	-0.61
A/gs	ETR	0.01	-0.02								
A/gs	NPQ	-0.42	-0.20								

Supplementary Figure S2. Variation in gas exchange and chlorophyll fluorescence parameters of cowpea subject to drought stress and rewatering. Data are expressed as log_2 means and represented using a correlation heatmap. Measurements were taken every two days (with the exception of D2) for 12 d. For the dataset used and interpretation of abbreviations, see Supporting Information Table S4. (**A**) Parameters with similar Pearson *r* values were grouped by two-way HCA, based on complete agglomeration. (**B**) Parameters with shared and divergent responses; *r* values are highlighted for $P < 0.01$ (yellow); $P < 0.05$ (red); and $P > 0.05$ (grey).

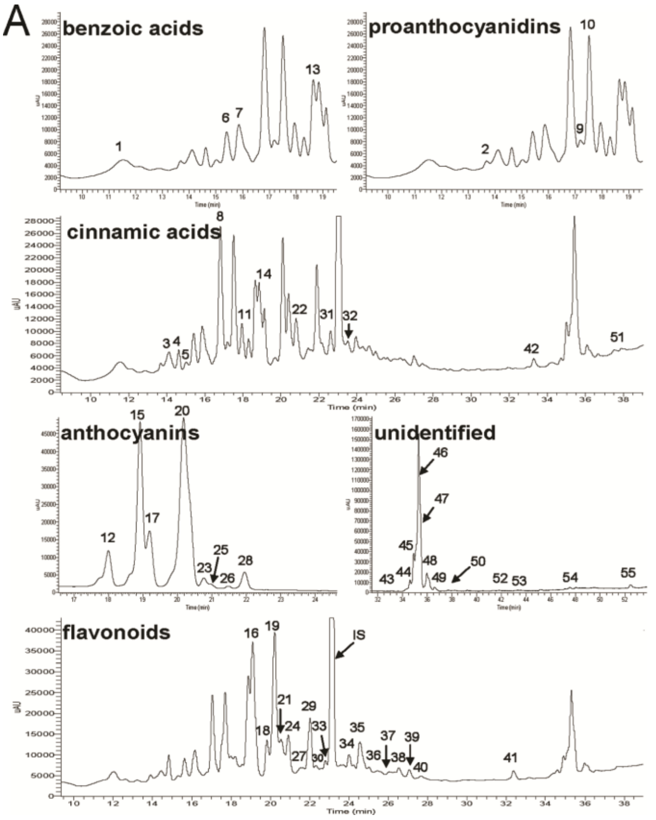

B

No	Retention time	λ absorption	Compound	Abbreviation
1	11.54	280 nm	Gallic acid	gallic
2	13.62	290 nm	Catechin 3-*O*-glucoside	cat-3-glc
3	14.12	320 nm	*trans-p*-Coumaroylaldaric acid 1	cou-ald1
4	14.62	320 nm	*trans-p*-Coumaroylaldaric acid 2	cou-ald2
5	15.41	320 nm	*trans-p*-Coumaroylaldaric acid 3	cou-ald3
6	15.86	280 nm	Protocatechuic acid	protocatec
7	16.82	280 nm	Hydroxybenzoic acid	hydroxyben
8	17.20	320 nm	*trans*-Feruloylaldaric acid 1	fer-ald1
9	17.51	290 nm	Catechin	catechin
10	17.94	290 nm	Catechin 7-*O*-glucoside	cat-7-glc
11	18.00	320 nm	*trans*-Feruloylaldaric acid 2	fer-ald2
12	18.11	520 nm	Delphinidin 3-*O*-galactoside	del-3-gal
13	18.65	280 nm	Vanillic acid	vanillic
14	18.83	320 nm	Caffeic acid	caffeic
15	18.92	520 nm	Delphinidin 3-*O*-glucoside	del-3-glc
16	19.12	360 nm	Quercetin 3,7-*O*-diglucoside	que-3.7-dglc
17	19.19	520 nm	Cyanidin 3-*O*-galactoside	cya-3-gal
18	19.89	360 nm	Quercetin 3-*O*-diglucoside-4-*O*-glucoside	que-3.4-dglc
19	20.18	360 nm	Myricetin 3-*O*-diglycoside	myr-3-dglc
20	20.22	520 nm	Cyanidin 3-*O*-glucoside	cya-3-glc
21	20.56	360 nm	Quercetin 3-*O*-diglycoside	que-3-dglc
22	20.77	320 nm	Coumaric acid	coumaric
23	20.79	520 nm	Petunidin 3-*O*-galactoside	pet-3-gal
24	20.91	360 nm	Quercetin 3-*O*-arabinosyldiglucoside	que-3-adglc
25	21.02	520 nm	Peonidin 3-*O*-glucoside	peo-3-glc
26	21.49	520 nm	Petunidin 3-*O*-glucoside	pet-3-glc
27	21.67	360 nm	Myricetin 3-*O*-glucoside	myr-3-glc
28	21.95	520 nm	Malvidin 3-*O*-glucoside	mal-3-glc
29	22.01	360 nm	Kaempferol 3-*O*-diglycoside	kae-3-dglc
30	22.61	360 nm	Quercetin 7-*O*-galactoside	que-7-gal
31	22.64	320 nm	Ferulic acid	ferulic
32	23.41	320 nm	*trans*-Feruloyl-methylaldaric acid 1	fer-mal1
33	23.52	360 nm	Dihydroquercetin	dhyd-que
**IS**	**23.76**	**280 nm**	**Internal standard (naringin)**	**IS**
34	24.04	360 nm	Quercetin 3-*O*-galactoside	que-3-gal
35	24.56	360 nm	Quercetin 3-*O*-glucoside	que-3-glc
36	25.02	360 nm	Quercetin 3-*O*-6″-malonylglycoside	que-3.6-mglc
37	26.04	360 nm	Quercetin 3-*O*-(6″-feruloyl)-diglycoside	que-3.6-fdglc
38	26.54	360 nm	Quercetin 3-*O*-(6″-diacetyl)-diglycoside	que-3.6-ddglc
39	27.06	360 nm	Quercetin 3-*O*-(6″-succinoyl)-glycoside	que-3.6-sglc
40	28.95	360 nm	Quercetin	quercetin
41	32.37	360 nm	Kaempferol 3-*O*-glucoside	kae-3-glc
42	33.29	320 nm	*trans*-Feruloyl-methylaldaric acid 2	fer-mal2
43	34.32	435 nm	Unknown 1	unknown1
44	34.65	435 nm	Unknown 2	unknown2
45	34.94	435 nm	Unknown 3	unknown3
46	35.18	435 nm	Unknown 4	unknown4
47	35.35	435 nm	Unknown 5	unknown5
48	36.12	435 nm	Unknown 6	unknown6
49	36.71	435 nm	Unknown 7	unknown7
50	37.55	290 nm	Unidentified 1	uniden1
51	37.88	320 nm	*trans-p*-Coumaroylaldaric acid 4	cou-al4
52	41.94	290 nm	Unidentified 2	uniden2
53	43.51	290 nm	Unidentified 3	uniden3
54	47.28	290 nm	Unidentified 4	uniden4
55	52.74	290 nm	Unidentified 5	uniden5

Supplementary Figure S3. High-performance liquid chromatography separation of secondary metabolites in cowpea. (**A**) Chromatograms are arranged into broad classes; for the different classes, a number was assigned to each metabolite. All chromatograms were acquired from a representative drought-stressed Pinhel leaf; anthocyanins scarcely accumulated in the leaves and roots of Fradel or Pinhel; and therefore were acquired from another cultivar. (**B**) List of 55 compounds for which cowpea was analysed, sorted by the first column according to numbers in the chromatograms. Most compounds were identified from comparison with a purified standard, whereas a few were identified solely based on literature and databases.

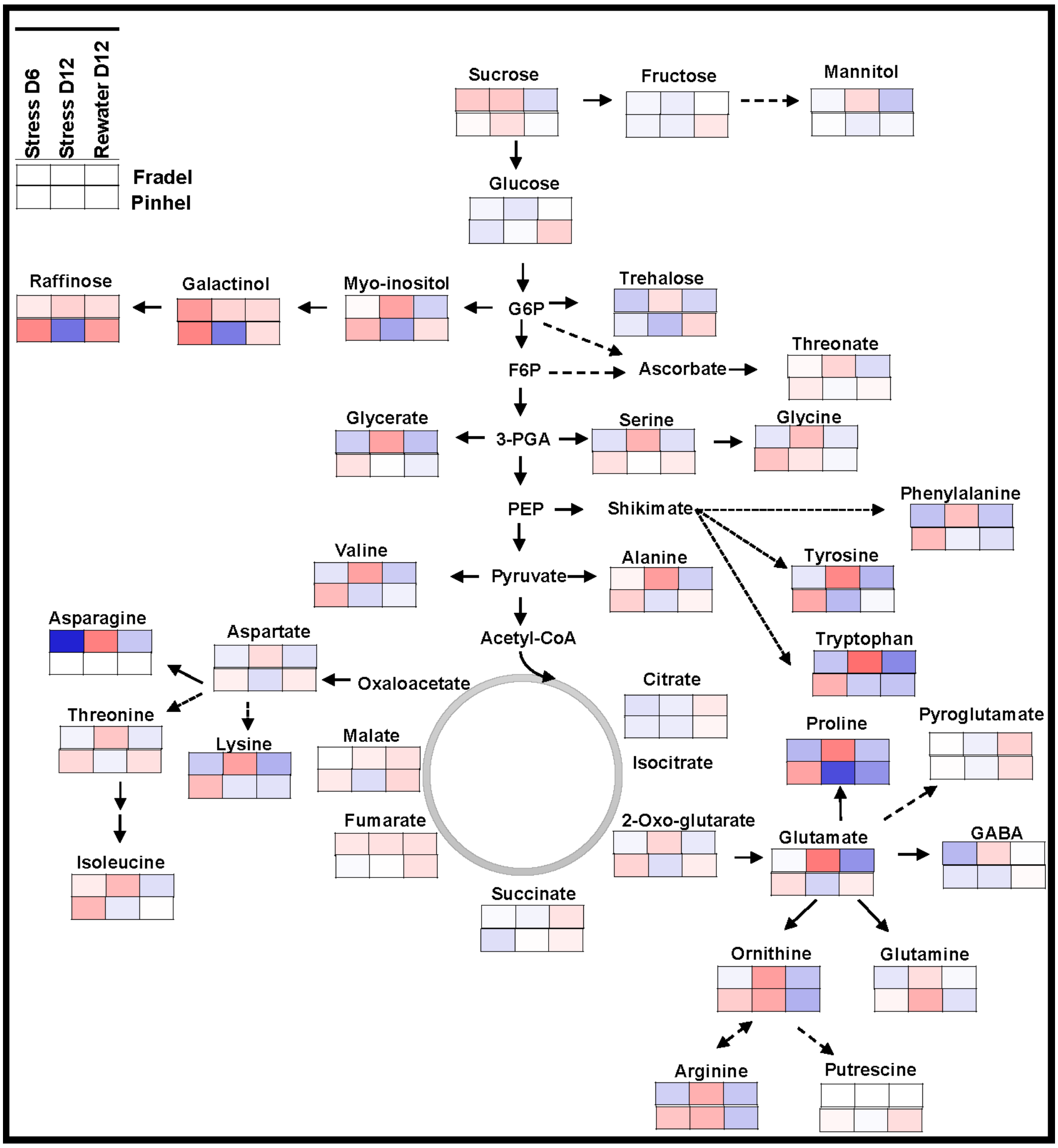

Supplementary Figure S4. Mapping of root metabolites in representative pathways. Metabolites are visualised using the averaged log fold-change ratios between drought-stressed and well-watered plants (n = 4 or 6) harvested at day 6 and 12, and between rewatered and drought-stressed plants harvested at day 12. Fradel = top row cells; Pinhel = bottom row cells. Red and blue indicate increased and decreased levels, respectively.

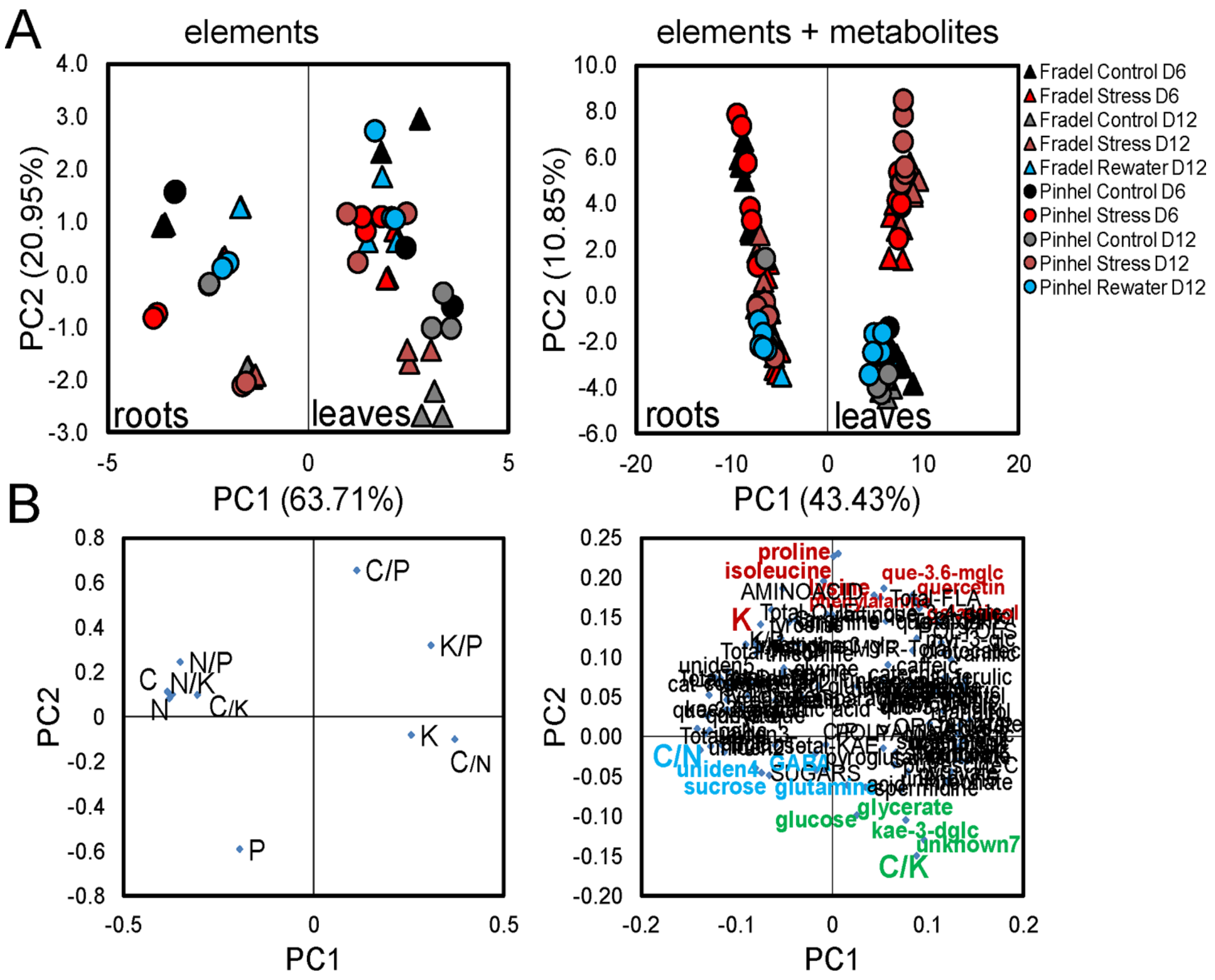

Supplementary Figure S5. Separation of elemental and organic solutes according to drought and rewatering treatments using PCA; the separation is based on the levels of compounds in well-watered control (black and grey for 6 and 12 d of stress, respectively), drought-stressed (red and brown for 6 and 12 d of stress, respectively), and rewatered (blue for plants stressed for 6 d and rewatered for 6 d) plants (n = 4 or 6). (**A**) Scores of PC1 and PC2 are shown for the square root-transformed values with pareto scaling of 4 elements and their stoichiometry ratio and of 88 metabolites and their sums; (**B**) PCA loadings with highest positive and negative values (Supporting Information Table S6) associated with metabolic responses to stress/rewatering are tagged in red and green, respectively. Loadings associated with other factors, e.g., sampling time, cultivar, or organ, are highlighted in blue. Metabolites were grouped according to functional categories, indicated in capital letters. The complete names of secondary metabolites can be retrieved from Supporting Information Table S3.
